# Transcriptome analysis in tissue sectors with contrasting crocins accumulation provides novel insights into apocarotenoid biosynthesis and regulation during chromoplast biogenesis

**DOI:** 10.1038/s41598-018-21225-z

**Published:** 2018-02-12

**Authors:** Oussama Ahrazem, Javier Argandoña, Alessia Fiore, Carolina Aguado, Rafael Luján, Ángela Rubio-Moraga, Mónica Marro, Cuauhtémoc Araujo-Andrade, Pablo Loza-Alvarez, Gianfranco Diretto, Lourdes Gómez-Gómez

**Affiliations:** 1 Instituto Botánico, Departamento de Ciencia y Tecnología Agroforestal y Genética, Facultad de Farmacia, Universidad de Castilla-La Mancha, Campus Universitario s/n, 02071 Albacete, Spain; 2 Facultad de Ciencias Ambientales y Bioquímica, Universidad de Castilla-La Mancha, Campus Tecnológico de la Fábrica de Armas, Av. Carlos III s/n, 45071 Toledo, Spain; 3 Italian National Agency for New Technologies, Energy, and Sustainable Development, Casaccia Research Centre, 00123 Rome, Italy; 4 Synaptic Structure Laboratory, Instituto de Investigación en Discapacidades Neurológicas (IDINE), Departamento Ciencias Médicas, Facultad de Medicina, Universidad Castilla-La Mancha, Campus Universitario s/n, 02071 Albacete, Spain; 5 ICFO-Institut de Ciencies Fotoniques, The Barcelona Institute of Science and Technology, Av. Carl Friedrich Gauss 3, 08860 Castelldefels, Spain

## Abstract

Crocins, the red soluble apocarotenoids of saffron, accumulate in the flowers of Crocus species in a developmental and tissue-specific manner. In *Crocus sieberi*, crocins accumulate in stigmas but also in a distinct yellow tepal sector, which we demonstrate contains chromoplast converted from amyloplasts. Secondary metabolites were analysed by LC-DAD-HRMS, revealing the progressive accumulation of crocetin and crocins in the yellow sector, which were also localized *in situ* by Raman microspectroscopy. To understand the underlying mechanisms of crocin biosynthesis, we sequenced the *C. sieberi* tepal transcriptome of two differentially pigmented sectors (yellow and white) at two developmental stages (6 and 8) by Illumina sequencing. A total of 154 million high-quality reads were generated and assembled into 248,099 transcripts. Differentially expressed gene analysis resulted in the identification of several potential candidate genes involved in crocin metabolism and regulation. The results provide a first profile of the molecular events related to the dynamics of crocetin and crocin accumulation during tepal development, and present new information concerning apocarotenoid biosynthesis regulators and their accumulation in *Crocus*. Further, reveals genes that were previously unknown to affect crocin formation, which could be used to improve crocin accumulation in Crocus plants and the commercial quality of saffron spice.

## Introduction

Carotenoids are C40 polyene lipophilic compounds derived from the isoprenoid biosynthetic pathway. These secondary metabolites act as precursors of apocarotenoids, which are widely present in all living organism and with diverse biological functions^1^. Certain apocarotenoids confer bright colours to the tissues in which they accumulate, as crocins and bixin, which are present at high levels in certain tissues of *Crocus sativus* and *Bixa orellana*, respectively^2,3^. The biosynthesis and accumulation of these apocarotenoids is of great interest due to their economic value, antioxidant properties and their potential impact on human health^4,5^.

The majority of the genes encoding the enzymes of the carotenoid pathway have been characterized in higher plants, and the presence of several isoforms for most of the carotenogenic genes suggest a complex regulation of the pathway, in addition to the further specialization of such pathway in certain rich-chromoplast tissues^6^. Recent research has demonstrated that the regulation of chromoplast biogenesis plays a crucial role in controlling carotenoid content by enabling great biosynthesis and high storage capacity^7–10^. Inside the chromoplast, carotenoids are stored in diverse suborganellar structures, which can be globulous, membranous, tubulous, or crystalline^11^. Carotenoids are the substrates of carotenoid cleavage dioxygenase (CCD) enzymes, producing apocarotenoids that play diverse functions as bioactive molecules^12^. In plants, CCD showed cytosolic or plastid locations^1^, suggesting different compartments for apocarotenoid biosynthesis and accumulation. The apocarotenoid crocetin and its glucosylated derivatives, crocins, are yellow pigments present in the flowers of almost all *Crocus* species^5,13,14^. The biosynthetic pathway for carotenoids and crocetin begins with the conversion of geranyl-geranyl pyrophosphate to phytoene, which is the first enzymatic step in the carotenoid biosynthetic pathway, followed by the action of phytoene desaturase (PDS), the subsequent enzyme for the desaturation of phytoene. In plants, this non-coloured carotenoid is transformed in the red linear carotenoid lycopene following desaturation and isomerization reactions catalysed by at least three enzymes (ζ-carotene isomerase, Z-ISO; ζ-carotene desaturase, ZDS; carotenoid isomerase, CrtISO)^6^. Lycopene acts as substrate for plant β- and ε-cyclase enzymes (LCYB and LCYE, respectively). Cyclization of lycopene yields β-carotene via the action of LCYB. On the other side, the sequential activity of LCYE and LCYB forms α-carotene, leading to the synthesis of lutein, while the β-carotene hydroxylase (BCH) catalyses the formation of the yellow carotenoid zeaxanthin from β-carotene^15^. Zeaxanthin is the direct precursor for the biosynthesis of crocetin, mediated by the action of the carotenoid cleavage dioxygenase CCD2 in Crocus species^16–18^ and CCD4 related enzymes in *Buddleja davidii*^19^. Both enzymes recognize and cleave zeaxanthin at 7, 8, 7′, 8′ double bonds, producing two molecules of the volatile 2,6,6-trimethyl-4-hydroxy-1-carboxaldehyde-1-cyclohexene (HTCC), the picrocrocin precursor, and one molecule of crocetin-dialdehyde. Crocetin is further glucosylated to produce crocins with different numbers of attached glucose molecules^20^. Although crocetin is produced inside the chromoplast^18^, crocins accumulate in the vacuoles of saffron stigmas at high levels, forming crystal structures^21^. Crocins’ biosynthesis and accumulation are developmentally regulated in Crocus species, increasing their concentration during the initial stages of development^22,23^. Little information is available on the regulation and accumulation of these soluble apocarotenoids during the development, or the implications, if any, of chromoplast biogenesis during this process. In this work, chromoplast structure, secondary metabolites location, metabolomics, and transcriptomic analyses were carried out to understand the regulation of the metabolic and structural changes occurring in *Crocus sieberi* tepal plastids during the transformation of amyloplasts into chromoplasts, the relationship with biosynthesis, and regulation of crocins’ accumulation. Our analysis completes the recently published saffron transcriptomes data set, which provides gene expression data for different tissues and mature stigma. Thus, the transcriptome of the key stages in apocarotenoid deposition in contrasting sectors of the tepal tissue of an additional *Crocus* species is now available, and shows that the apocarotenoid metabolism and the gene transcript levels are already perturbed at very early developmental stages in *Crocus* and are associated with chromoplast development.

## Results and Discussion

### Developmental and tissue-specific accumulation of apocarotenoids in *C. sieberi* tepals

**Figure 1:**
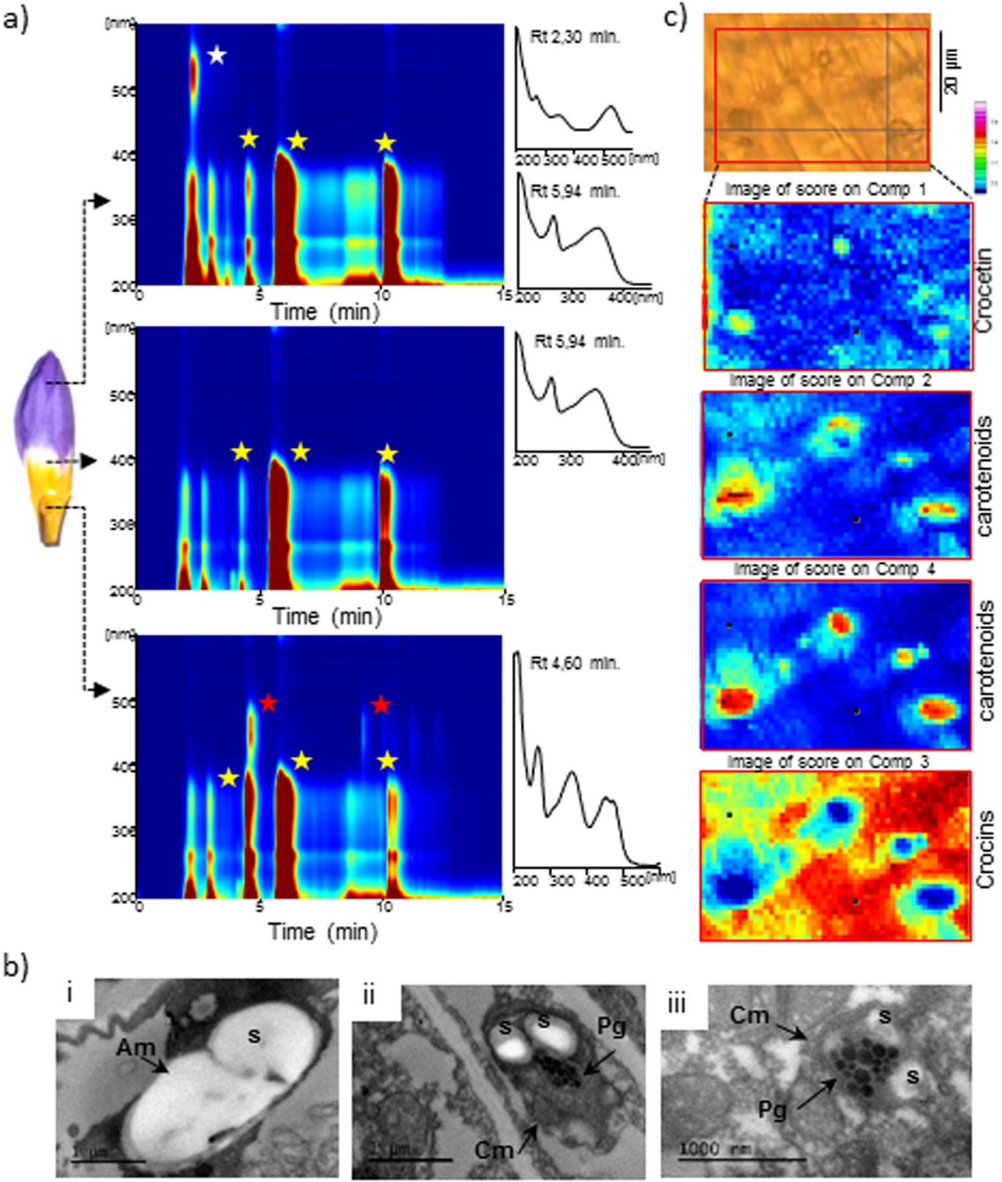
Differential accumulation and localization of crocins in *C. sieberi* flowers and the plastids ultrastructure. (**a**) *C. sieberi* flowers at preanthesis stage are shown. The tepals present three distinctive colourations due to the accumulation of different pigments as shown in the LC-PDA/UV isoplot of the right side. (**b**) TEM images showing the amyloplast and chromoplast in white sector (i) and yellow sector (ii,iii). Am, amyloplast; Cm, chromoplast; Pg, plastoglobules; S, starch. (**c**) RAMAN analyses over the yellow sector from tepals at preanthesis. The colours of the map describe the integral Raman intensity.

Previous analytical data of tepals’ aqueous extracts from *C. sieberi* indicated the presence of several crocin types, with crocetin as the aglycon and between 6–14 units of glucose associated with the crocetin skeleton^13^. The tepals of *C. sieberi* ssp. *sublimis* tricolour present a characteristic colour pattern, with three well differentiated sectors (Fig. 1a). The uppermost part of the tepal presents a lilac colour due mainly to the presence of delphinidin 3,5-β-d-diglucoside and very low amounts of petunidin 3,5-β-d-diglucoside (Fig. 1a)^24^. Just below this lilac region, the white sector of the tepal contains flavonoids, showing a similar pattern to the one present in the lilac region but also to the one observed for the yellow part (Fig. 1a). These flavonoids have been identified previously as quercetin 3-*O*-β-sophoroside, kaempferol 3-*O*-β-sophoroside, kaempferol 3-*O*-α-(2-*O*-β-glucosyl)-rhamnoside-7-*O*-β-(6-*O*-malonyl)-glucoside, kaempferol 3-*O*-α-(2,3-di-*O*-β-glucosyl) rhamnoside, kaempferol 3-*O*-α-(2-*O*-β-glucosyl) rhamnoside-7-*O*-β-(6-*O*-acetyl)glucoside and kaempferol 3-*O*-α-(2-*O*-β-glucosyl)-rhamnoside^24^. The basal part of the tepal is characterized by a bright yellow colour, due to the accumulation of crocins (Fig. 1a)^13^.

Transmission electron microscopy (TEM) was used to examine the plastid morphology in the white and yellow sectors of flowers at preanthesis. Chromoplast development may involve transitions from other type of plastids such as amyloplasts, leucoplasts or chloroplasts^25^. Amyloplasts with large starch granules of different sizes were observed in the white sectors (Fig. 1b–i). By contrast, the samples from the yellow sector showed the presence of globular chromoplasts that contain round plastoglobuli varying in number, size, and electron density, while only low amounts of starch grain remnants were detected (Fig. 1b–ii/iii).

By *in situ* Raman confocal microscopy, we were able to image with subcellular resolution the concentration and localization of crocins, crocetin and carotenoids in the cells of the yellow part of the tepals at preanthesis. Multivariate methods were used to convolve pure molecular components associated with crocetin, crocins and carotenoids (Fig. 1c) from the Raman spectra obtained in each image. Component 1 can be assigned to crocetin, as manifested by the presence of a band approximately 1533 cm^−1^. Components 2 and 4 can be assigned to carotenoids by the presence of bands at 1522 and 1527 cm^−1^ ^26^, and component 3 can be assigned to crocins by the presence of a band around 1535 cm^−1^. A molecular database was previously constructed measuring the Raman spectra of pure crocins and crocetin, and the bands coincide with these assignments (Fig. S1). By this technique, we were able to establish that the chromoplasts contain crocetin as a dominant apocarotenoid, while crocins accumulate in the vacuole, as shown in Fig. 1c.

**Figure 2:**
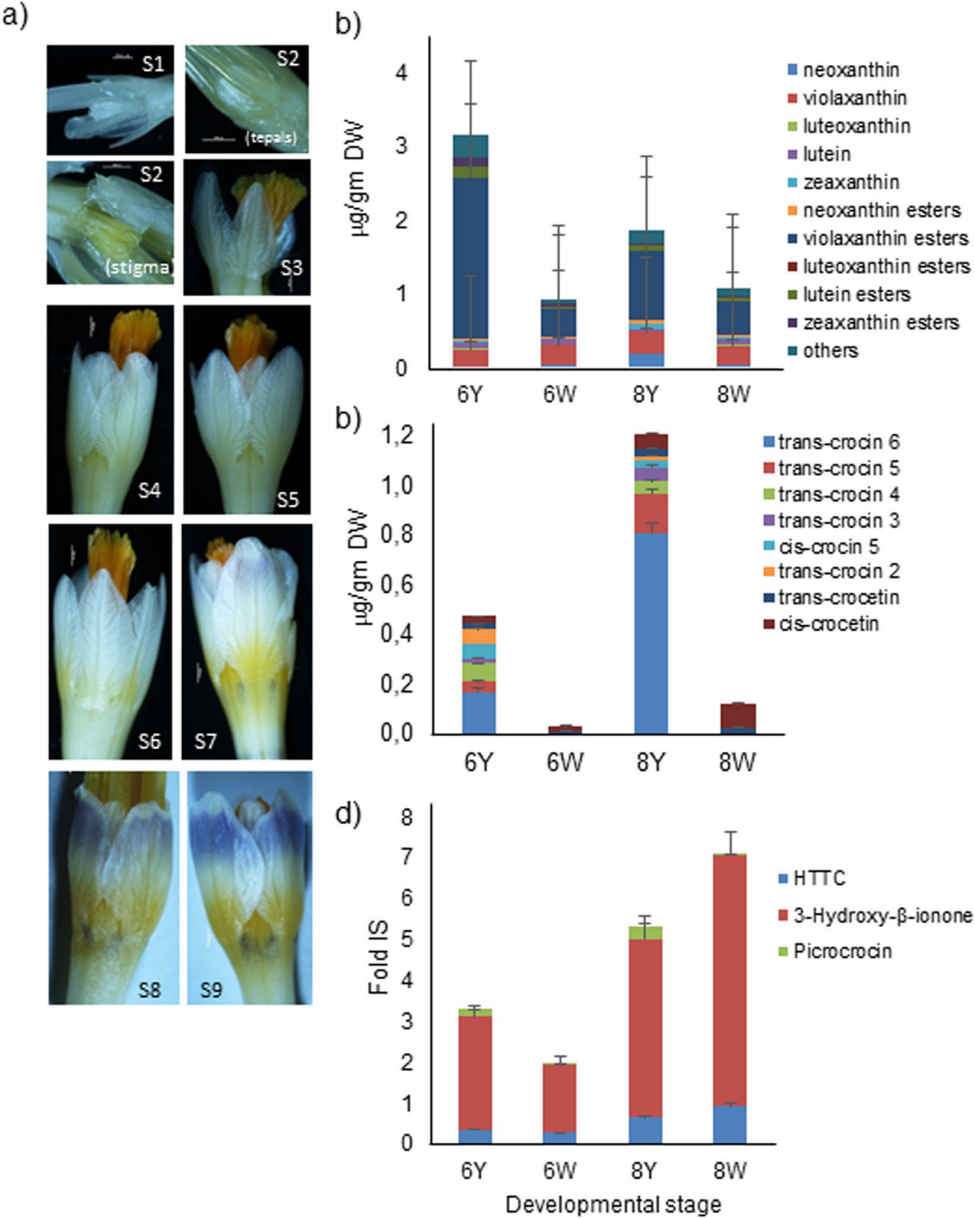
Definition and characterization of RNA-seq experimental material in *Crocus sieberi*, and changes in carotenoids and apocarotenoids levels in white and yellow sectors of two different developmental stages of *C. sieberi* tepals. (**a**) Images showing the development of tepals in *C. sieberi* flowers and the increasing tepal colouration associated with each developmental stage (S1–S9). (**b**) Levels of carotenoids. (**c**) Levels of crocetin and crocins. D, Levels of picrocrocin, HTTC and 3-hydroxy-β-ionone. The yellow colouring part of tepals at S6 (6Y) and stage S8 (8Y), and the white part of tepals at stage S6 (6W) and stage S8 (8W).

Tepal development in *C. sieberi* is characterized by increasing colouration of the basal and upper parts (Fig. 2a). Apocarotenoid deposition in the basal part of the tepal occurs before the accumulation of anthocyanins in the upper part of the tepal (Fig. 2a). The yellow and lilac colouration increased until the flower is fully developed (Figs 1a and 2a). For this study, nine different stages of flower development in *C. sieberi* were designated S1-S9, all of them before anthesis. Stage 1 (S1) is characterized by the presence of small white stigma and tepals (Fig. 2a). In the following stage, S2 (Fig. 2a), the stigma showed a pale yellow colouration, with no perceptible changes in size and colour with respect to S1. In Stage 3 (S3), the tepals increased in size but maintained their white colour, while the stigma increased in size and colour, showing a bright orange colour. In Stage 4 (S4), the tepals and stigma increased in size. In Stage 5 (S5), the stigma and tepal continued growing, while in Stage 6 (S6), a pale yellow colouration began to manifest in the base of the inner tepals. By Stage 7 (S7), all the tepals showed a yellow colouration at the base of the tissue, and a blue colouration began to be visible in the upper part of the tepals. At Stage 8 (S8), the lower part of the tepals was fully yellow and the lilac colouration was more evident in the upper part. From this stage, the colouration of the lower (yellow) and upper (lilac) parts of the tepals started to increase (S9, Fig. 2a) until reaching the stage of preanthesis (Fig. 1a).

### Carotenoid, apocarotenoid, and flavonoid content in selected developmental stages S6 and S8

Tissue dissection was performed using tepals from developmental stages S6 and S8. The presence of phenolic acids, flavonoids, carotenoids and apocarotenoids was evaluated in the yellow (6–8Y) and white (6–8W) parts of these two developmental stages by LC-HRMS analyses (Table 1 and Fig. 2). Among the phenolic acids, pyrogallol was the major compound detected in both developmental stages, with higher levels in the yellow part of tepals from stage S8. In the flavanone group, the flavonoid kaempferol was present at high levels in the white part of S6 (6W) and S8 (8W) developmental stages. Flavonoids were mainly present as glycosides, with the prevalence of quercetin and kaempferol derivatives (Table 1). Carotenoids were present at low levels, but their distribution was opposite to the one observed for flavonoids, with major levels in the yellow samples of both developmental stages (6Y and 8Y), and a prevalence of violaxanthin esters in its free form (Fig. 2b). Further, the levels of crocins and other apocarotenoids were also analysed (Fig. 2c,d). Crocetin and crocins were present at higher levels in the yellow sectors of both developmental stages, but at higher levels in 8Y than in 6Y. Interestingly, crocetin was detected in the white sectors of both developmental stages (6W and 8W), while crocins were not observed (Fig. 2c). The accumulation of crocins along with the development of tepals is also observed in the related species *Crocus ancyrensis*^23^, also characterized by the accumulation of highly glucosylated crocins in stigma and tepals^13^. This developmental accumulation of crocins is also present during the development of the saffron stigma^22^. Picrocrocin was also present at higher levels in the yellow sector (Fig. 2d); however, the apocarotenoid 3-hydroxy-β-ionone, resulting from the 9, 10; 9′, 10′ cleavage of zeaxanthin, was detected at higher levels in all the samples, and mainly accumulates in the 8W sample (Fig. 2d). This result could find an explanation considering that both CCD1 and CCD4 enzymes from different plants, including saffron, have been shown to catalyse the cleavage reaction resulting in the production of this apocarotenoid volatile^1^.

**Table 1:** Phenolic acids and flavonoids identified in *C. sieberi* tepals at different developmental stages.

Metabolite	Class	S6-yellow (6Y)		S6-white (6W)		S8-yellow (8Y)		S8-white (8W)	
		AVG	ST.DEV.	AVG	ST.DEV	AVG	ST.DEV	AVG	ST.DEV
Caffeic acid	Phenolic acids	0,0183	0,0016	0,0186	0,0032	0,0260	0,0015	0,0217	0,0030
Coumaric acid	Phenolic acids	0,0308	0,0018	0,0726	0,0120	0,0136	0,0018	0,1158	0,0106
Gallic acid	Phenolic acids	0,0041	0,0006	0,0115	0,0010	0,0104	0,0014	0,0225	0,0038
3-Hydroxy-4-methoxybenzoic acid	Phenolic acids	0,0824	0,0113	0,0540	0,0090	0,1208	0,0108	0,1549	0,0257
Protocatechuic acid methyl ester	Phenolic acids	0,0687	0,0049	0,0536	0,0056	0,1307	0,0202	0,1519	0,0127
Pyrogallol	Phenolic acids	1,9996	0,3085	1,0996	0,0883	2,8331	0,1945	2,0239	0,3361
Vanillic acid	Phenolic acids	0,0672	0,0046	0,0554	0,0019	0,1146	0,0154	0,1487	0,0156
Apigenin/Genistein	Flavanone	0,0000	0,0000	0,0135	0,0018	0,0025	0,0001	0,0078	0,0003
Kaempferol	Flavanone	0,4392	0,0493	19,4616	3,2886	1,0449	0,1406	23,0590	1,3124
Myricetin	Flavanone	0,0579	0,0079	0,0064	0,0007	0,1743	0,0238	0,0333	0,0045
Quercetin	Flavanone	0,3330	0,0190	0,0807	0,0062	0,5961	0,0431	0,0996	0,0080
Dihydrokaempferol 7-O-glucoside	flavanone glycosides	0,0338	0,0055	0,0430	0,0024	0,0478	0,0027	0,0919	0,0170
6-Hydroxyluteolin 7-glucoside	Flavanone glycosides	0,2574	0,0443	0,0822	0,0111	0,5831	0,0784	0,1521	0,0063
6 -Hydroxyluteolin 7-methyl ether 6-glucoside	Flavanone glycosides	0,1904	0,0111	0,3557	0,0485	0,2819	0,0385	0,5134	0,0292
6 -Hydroxyluteolin 7-rhamnosylglucoside	Flavanone glycosides	2,5986	0,2231	1,7405	0,1248	4,2637	0,3080	3,3141	0,4458
Isorhamnethin 3,4′-diglucoside	Flavanone glycosides	0,0263	0,0022	0,0148	0,0013	0,0128	0,0020	0,0000	0,0000
Kaempferol 3-β-D-glucopyranoside/Kaempferol 7-β-D-glucopyranoside	Flavanone glycosides	9,6030	0,5466	13,7021	2,1137	15,8665	1,0890	24,7732	1,7756
Kaempferol‐3‐O‐rutinoside/Apigenin 6,8-digalactoside	Flavanone glycosides	4,1894	0,7144	7,9433	0,5452	6,1200	0,4386	9,9973	0,5690
Kaempferol 3-rutinoside-7-glucoside	Flavanone glycosides	0,1902	0,0315	0,1429	0,0102	0,2303	0,0210	0,1583	0,0213
Kaempferol 7-sophoroside	Flavanone glycosides	47,7158	3,9831	28,7814	2,6229	73,7194	5,7009	61,3365	8,3710
Kaempferol 3-O-sophoroside-7-glucoside	Flavanone glycosides	0,0303	0,0050	0,4359	0,0337	0,0520	0,0030	0,6282	0,0450
Kaempferol 3,7,4′-triglucoside	Flavanone glycosides	0,1055	0,0110	0,1046	0,0060	0,1512	0,0203	0,1895	0,0158
Myricetin 3-glucosyl-(1- > 2)-rhamnoside-7-glucoside	Flavanone glycosides	0,1993	0,0178	0,1038	0,0140	0,2796	0,0464	0,0222	0,0033
Naringin	Flavanone glycosides	0,2796	0,0431	0,1396	0,0191	0,2632	0,0275	0,1418	0,0219
Naringenin 7-O-glucoside	Flavanone glycosides	0,1251	0,0086	0,0518	0,0037	0,1590	0,0128	0,0545	0,0049
Quercetin 3-diglucoside	Flavanone glycosides	1,5519	0,1200	0,3076	0,0510	2,8434	0,2199	0,5528	0,0853
Rhamnetin 3-rutinoside	Flavanone glycosides	0,0568	0,0076	0,0492	0,0041	0,0072	0,0007	0,0022	0,0001
Rutin	Flavanone glycosides	62,0546	10,2663	39,7265	6,5980	101,8356	18,8199	84,5190	6,1054

### Tissue dissection and transcriptome

To get insights about the regulatory mechanism involved in apocarotenoid deposition in the tepals of *C. sieberi*, the developmental stages S6 and S8 that showed contrasted colouration, along the development of tepals and apocarotenoids accumulation, were used for transcriptomic analyses. Four RNA samples of *C. sieberi*, including the yellow colouring part of tepals at S6 (6Y) and stage S8 (8Y), and the white part of tepals at stage S6 (6W) and stage S8 (8W) (Fig. 1d), were sequenced and analysed. A total of 190,385,616 high-quality reads (after removing of low quality and primer/adapter contaminated reads) ranging from 35 to 43 million for each sample were generated, with an average GC content (46.77%; Table 2). In general, all four libraries presented good quality, with an average of 98.83% of reads with base call quality at 99.9% probability (Q20) and 93.75% at 99.9% (Q30) (Table 2). The sequences were filtered for adaptors and sequencing artefacts, reducing the number of reads per library by 5.9 to 8.8% (high quality reads; Table 2) before transcriptome assembly. The assembled *C. sieberi* transcriptome contained 156,827,502 nucleotides in unigenes, with an average size of 632.12 bp, a N50 of 921 bp and a 42.92% GC content (Table 3). We compared the *de novo* transcriptome assembly of *C. sieberi* generated in our study with those obtained for *C. sativus*. The most recent published transcriptome over different tissues from *C. sativus* generated 112,037 transcripts with a largest average unigene length of 652 bp, a N50 length of 1031 bp, and %GC of 43%^27^, while the first published transcriptome, performed over flowers and stigma tissue, generated 64,438 transcripts with as average unigene length of 609 bp, a N50 length of 753 bp and a GC content of 43.99%^28^. Thus, the *de novo* transcriptome analyses generated from *C. sieberi*, were similar to those obtained from different tissues from saffron^27^.

**Table 2:** Data statistics of RNA-seq reads obtained from Illumina HiSeq-2000.

Sample	Total bases	High quality total bases (%)	Read Count	High quality read count (%)	GC(%)	Q20(%)	Q30(%)
S6-yellow (6y)	4,066,286,058	3,714,648,441 (91,3%)	40,260,258	38,085,176 (94,6%)	46,79	98,84	93,76
S6-white (6w)	4,490,317,590	4,229,865,363 (94,1%)	44,458,590	43,035,078 (96,8%)	46,96	98,84	93,8
S8-yellow (8y)	4,046,135,548	3,694,722,074 (91,3%)	40,060,748	37,905,510 (94,6%)	46,87	98,82	93,6
S8-white (8w)	3,814,108,450	3,480,777,267 (91,2%)	37,763,450	35,679,926 (94,5%)	46,47	98,85	93,8

**Table 3:** Statistics after merged assembly for all four analysed samples.

	All transcript contigs	Only longest isoform per “gene”
Total trinity ‘genes’	178,106	178,106
Total trinity transcripts	248,099	178,106
N50	921	647
Maximum contig length	12,010	12,010
Minimum contig length	201	201
Median contig length	387	330
Average contig length	632.12	523.4
Total assembled bases	156,827,502	93,220,589

### Functional annotation

**Figure 3:**
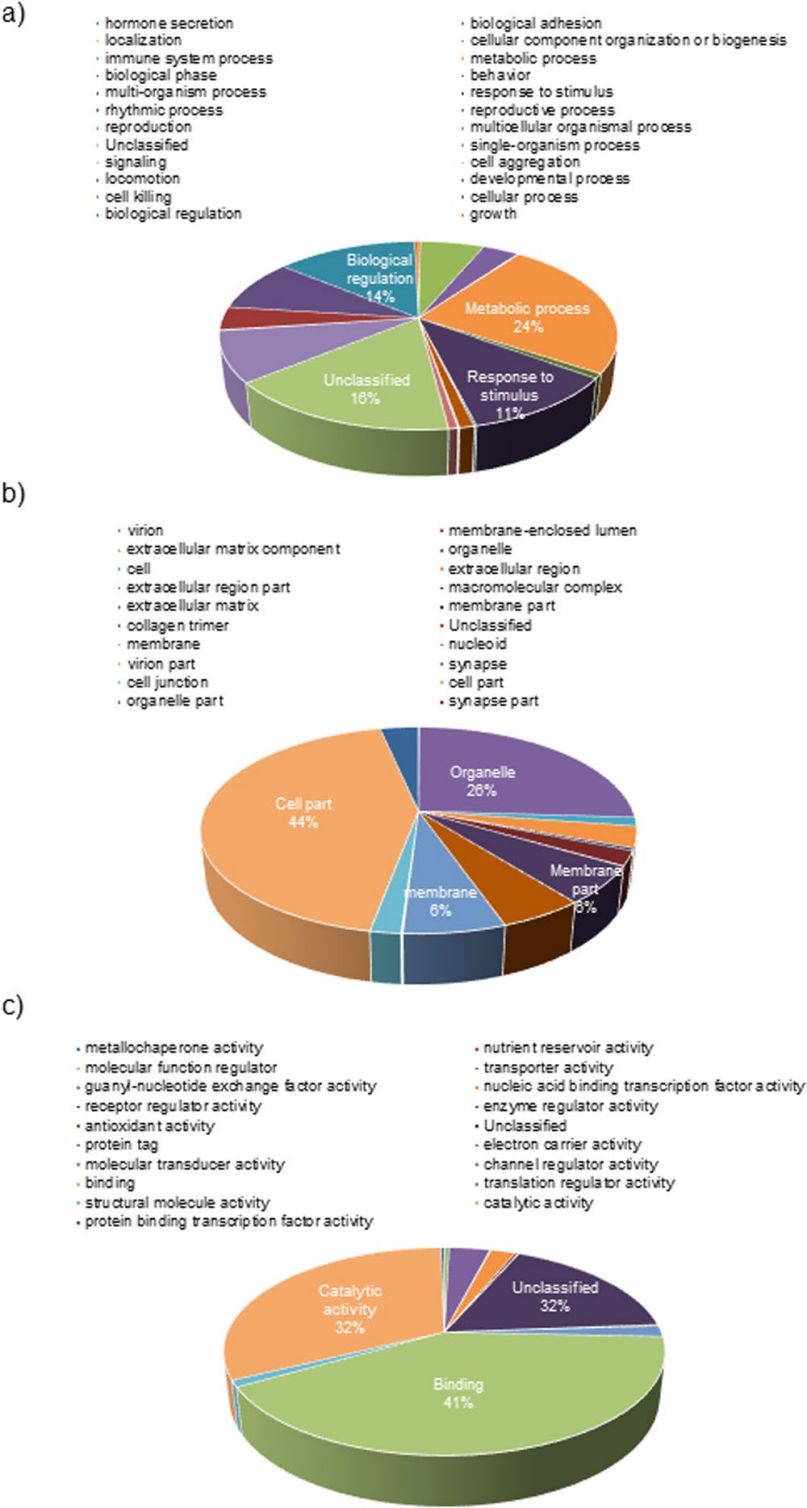
Gene ontology summary of all unigenes. The obtained unigenes were assigned to GO slim terms for biological processes (**a**), molecular functions (**b**), and cellular components (**c**). Numbers indicate percentages of each GO slim term within main ontologies.

The assembled *C. sieberi* transcriptome was used as a query for annotation by means of BLASTX searches based on sequence homologies in the databases of the National Center for Biotechnology Information (NCBI) non-redundant protein database (nr) using Blast2GO with an E-value cutoff of 1e^−06^. Gene ontology includes three main categories: Biological process, referring to the biological objective of the genes or gene products; Cellular components, referring to the place in the cell where the gene product is active; and Molecular function, defined by the biochemical activity of the genes or gene products^29^. Figure 3 illustrates the gene ontology annotation of the assembled unigenes from the *de novo* assembled transcriptome of *C. sieberi*. In total, 78,344 transcripts have been assigned with at least one GO Slim term under the biological process (30,438), molecular function (22,079) and cellular component (25,827) categories. Among the biological process terms, protein metabolism process (24.2%) was the most represented, followed by biological regulation (13.7%), response to stimulus (11%), cellular processes (9%), single-organism process (8.7%), localization (6.2%), developmental process (4%), and cell organization and biogenesis process (3.5%) (Fig. 3a). Figure 3b illustrates the cellular component category, which has a dominant subcategory of cell part (44%), organelle (26%), plasma membrane (6%) and other membranes (6%). Under molecular function, the term binding (41%) was most abundant, followed by catalytic activity (16%), transport activity (4%) and DNA or RNA binding (2.4%) (Fig. 3c). These annotations represent a profile for gene expression of *C. sieberi* tepals, suggesting that this species has diverse protein coding genes comprising their structural, regulatory, metabolic, and stress response mechanisms.

The conversion of mapped and assembled read counts into normalized digital transcript levels (Fragments Per Kilobase of exon per Million fragments mapped (FPKM)) allowed the determination of the 10 most abundant transcripts present in the tepal transcriptome (>5,000 FPKM values) (Table 4). Among them, the unigene c72319_g1_i1 was highly expressed in 8Y and showed identity with lipid transfer proteins (LTP), and 76% identity with an LTP isolated from stigmas of *C. sativus*, acting as an allergen^30^. LTPs can modulate the lipid composition and/or fluidity of membranes and regulate various cellular processes, including vesicular trafficking^31^. Unigene c67922_g1_i1, c65918_g2_i2, and c51370_g1_i1 showed identity to late embryogenesis abundant proteins (LEA), which are strongly associated with abiotic stress responses and are mostly induced by ABA (reviewed in^32^). The high expression levels of LEA transcripts in these samples could reflect the requirement for chilling to break flower bud dormancy in *C. sieberi*, as observed in other flowers buds^33^. Unigene c55171_g1_i1 was highly expressed in all the transcriptomes and showed identity to Bowman-Birk type proteinase inhibitors (BBI). BBI genes are highly expressed in those tissues susceptible to attack by predators or pathogens, e.g., rehydrated embryo, young leaves, root caps, and flower organs^34^; therefore, the higher expression of BBIs in tepals could serve as an effective protection mechanism against predators’ attacks. Unigene c58230_g1_i1 also showed identity to protease inhibitors (PI), but it was present at lower levels than c55171_g1_i1. In agreement with the other highest expressed transcripts, PIs also play an important role in plant stress responses (reviewed in^35^).

**Table 4:** Ten most abundant transcripts in the *C. sieberi* tepal transcriptome. The italic/bold correspond to the value of FPKM, ranging from italic (lower expression) to bold (higher expression).

Contig	FPKM				Best match identity
	6Y	6W	8Y	8W	
c72319_g1_i1	**5440,17**	**4470,47**	**10016,73**	**6593,94**	TAIR|locus:2081840 - symbol:LTP12 “lipid transfer protein 12” species:3702 “*Arabidopsis thaliana*” Uniprot:Q9SCZ0
c67922_g1_i1	**4125,84**	**5980,02**	*1668,08*	**4819,45**	UNIPROTKB|A3AHG5 - symbol:LEA1 “Late embryogenesis abundant protein 1” species:39947 “*Oryza sativa* Japonica Group”
c55171_g1_i1	**7297,16**	**6309,96**	**7608,11**	**7185,66**	UNIPROTKB|P01060 - symbol:BBI “Bowman-Birk type proteinase inhibitor 2” species:3885 “*Phaseolus vulgaris*” Uniprot:P01060
c58230_g1_i1	**4544,39**	**4711,95**	**3118,34**	**3378,65**	TAIR|locus:2051668 - symbol:LCR69 “AT2G02100” species:3702 “*Arabidopsis thaliana*” Uniprot:Q39182
c65918_g2_i2	**3559,21**	**3101,85**	*1485,41*	*2709,21*	TAIR|locus:2034376 - symbol:COR47 “AT1G20440” dehydrin species:3702 “*Arabidopsis thaliana*” Uniprot:P31168
c51370_g1_i1	**3024,32**	**3680,94**	*1102,45*	*2593,74*	TAIR|locus:2085171 - symbol:AT3G53040 “AT3G53040” Peamaclein species:3702 “*Arabidopsis thaliana*” Uniprot:Q9LF88
c69730_g1_i1	*1951,77*	*1970,74*	*2204,11*	**3387,94**	UNIPROTKB|Q9FUB7 - symbol:Q9FUB7 “Chalcone synthase” species:140968 “*Hypericum androsaemum*” Uniprot:Q9FUB7
c54813_g2_i1	*2259,96*	*2178,06*	*2573,75*	*1552,06*	TAIR|locus:505006706 - symbol:AT5G59845 “AT5G59845” species:3702 “*Arabidopsis thaliana*” Uniprot:Q8LFM2
c73954_g2_i1	*1457,81*	*1343,3*	*1759,61*	*1444,42*	TAIR|locus:2130883 - symbol:TIP2;2 “tonoplast intrinsic protein 2;2” species:3702 “*Arabidopsis thaliana*” Uniprot:Q41975
c73842_g3_i1	*1118,7*	*1651,43*	*1170,65*	*1928,86*	TAIR|locus:2117939 - symbol:PIP1;5 “AT4G23400” species:3702 “*Arabidopsis thaliana*” Uniprot:Q8LAA6

**Figure 4:**
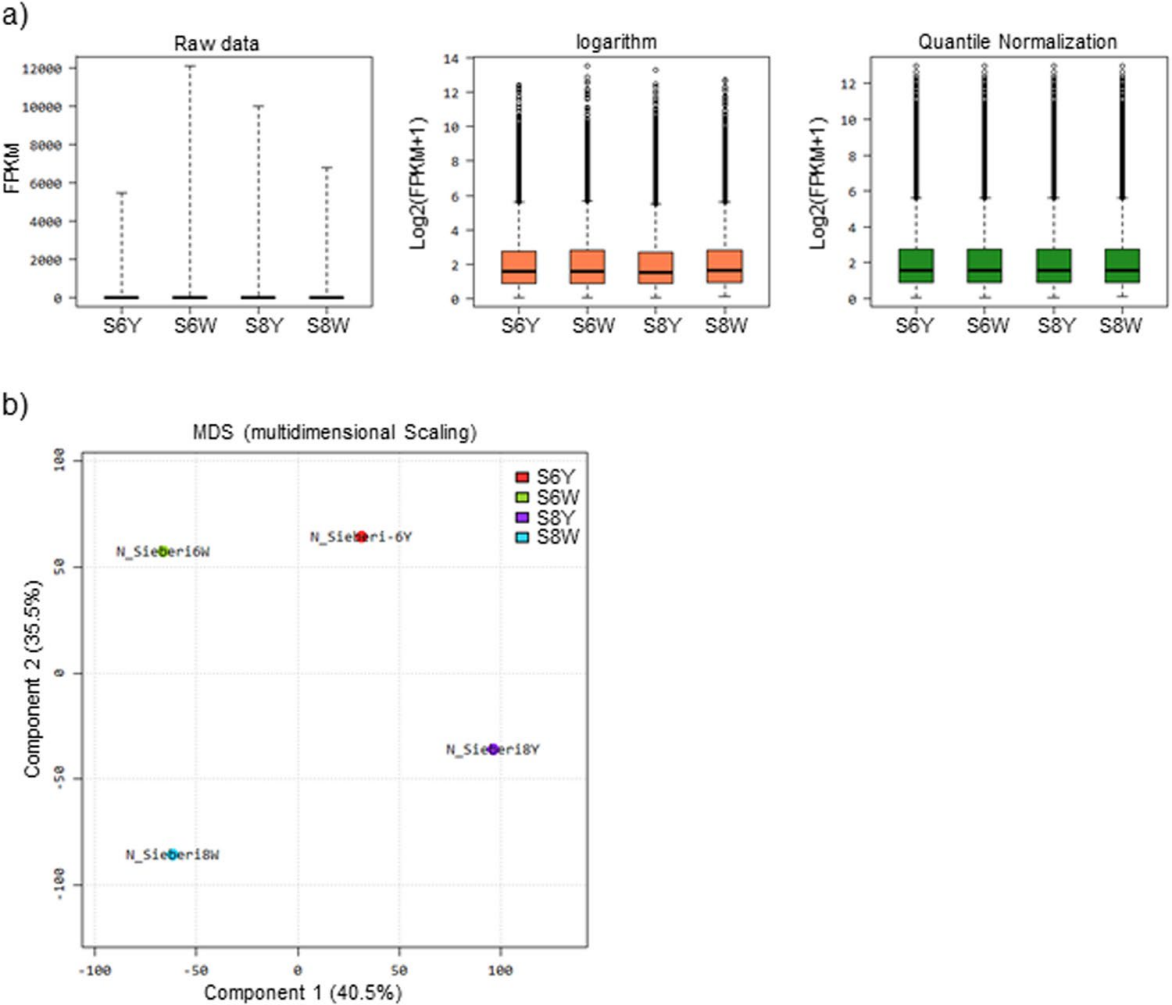
Differential expression analyses of *C. sieberi* tepal transcriptomes. (**a**) boxplots show before and after Raw Signal (FPKM + 1) Log2 transformation, before and after Quintile Normalization and corresponding samples’ expression scatter based on centile, median, 50 percentile, 75 percentile, maximum and minimum. (**b**) Multidimensional Scaling Plot for the obtained transcriptomes. Multidimensional Scaling Plot (MDS) is designed to indicate sample relationship similarity.

**Figure 5:**
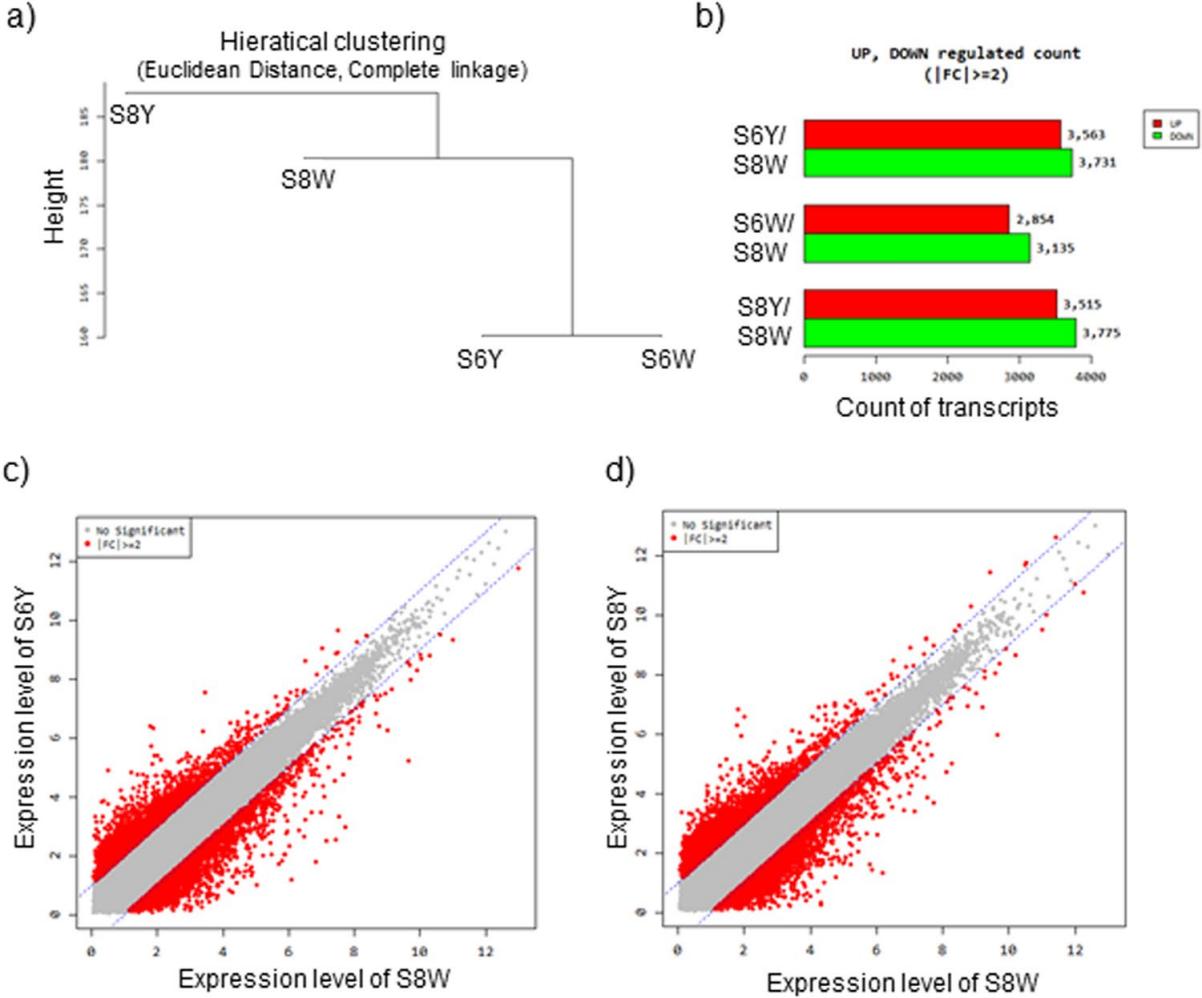
Differentially expressed genes (DEGs) detected between the analysed tissue sections. (**a**) Using each sample’s Log2 (FPKM + 1) value, the expression similarities were grouped together. (Distance metric = Euclidean distance, Linkage method = Complete linkage). (**b**) Changes in gene expression profiles between white and yellow samples in the selected developmental stages. The numbers of upregulated and downregulated genes are revealed by red and green, respectively. Differential expression analysis reveals more downregulation than up regulation. (**c**) The plot depicts fold change vs. mean expression of 6Y vs 8W. Points depict genes, with red indicating those genes that show significant differential expression. (**d**) The plot depicts fold change vs. mean expression. Points depict genes, with red indicating those genes that show significant differential expression of 8Y vs 8W.

Differential expression analyses were made using the EdgeR package^36^. Normalization was applied to the sample types to provide accurate differential expression rather than individual quantification. The distribution of the normalized expression level is shown in Fig. 4a. The EdgeR package adjusted the analysis, taking into account sequencing depths represented by library sizes. Variations between the samples from transcriptome analysis were used to produce a multidimensional scaling (MDS) plot to check for variations among samples (Fig. 4b), which allows the discovery of evidence of the spatial configuration to show how similar or dissimilar the tepal samples are. The plot indicates that 35.5% of differences among samples are due to the developmental stage, while the 40.5% of differences in gene expression are due to the colour or the presence or absence of apocarotenoids in the analysed samples. Further, hierarchical clustering of differentially expressed genes (DEG) showed relatively similar patterns of expression similarities between the 6W and 6Y (Fig. 5a), but clear differences with 8Y (Fig. 5). These data are in agreement with the variations observed at the metabolite pool levels present in the samples analysed from the two developmental stages (Fig. 2b).

The differential gene expression analysis resulted in a total of 15,038 significantly differentially expressed transcripts (p < 1e^−10^) among the analysed samples: 7,294 differentially expressed transcripts between 6Y and 8W 5,989 differentially expressed transcripts between 6W and 8W and 7,290 differentially expressed transcripts between 8Y and 8W (Fig. 5b). These results suggest substantial transcriptional differences related to the apocarotenoids content of the analysed samples but also associated with the developmental stage and further to flavonoid accumulation in the white samples (Fig. 5c).

### Up- and downregulated transcripts in apocarotenoids containing tepal tissue

We used the quantitative expression analysis of all four samples to investigate genes that are transcriptionally upregulated (Log2 [fold change (FC)] ≥ 2.0) and downregulated (Log2 [FC] ≤ −2.0) in the yellow samples in both developmental stages compared with white samples in the developmental stage S8 (8W). A total of 3,516 transcripts were upregulated in the 8Y sample compared with the 8W samples, and 3,774 were downregulated. Similar numbers were found in 6Y compared with 8W, with 3,564 transcripts upregulated and 3,730 downregulated. We also compared the transcripts upregulated and downregulated in 6W/8W, which were used to determine the exclusive transcripts upregulated in 6Y/8W and 8Y/8W, and therefore more probably involved in apocarotenoid biosynthesis. In this way, a total of 812 transcripts were found to be exclusively upregulated in the yellow stages (6Y and 8Y) (Fig. S3a). The 812 upregulated transcripts were grouped based on the cellular component category. The nucleus (33.8%) was followed by cytoplasm (30%), plasma membrane (10.8%), and extracellular and cell wall (9.7%) (Fig. S3b).

The analyses of the first 100 unigenes in 8Y/8W with Log2 [fold change (FC)] ≥ 4.1 (Table S1) reveals that the unigenes with the highest fold change were c75947_g2_i1/i2/i4 encoding for CCD2, catalysing the formation of crocetin from zeaxanthin in *Crocus* species^17,18^, followed by c54768_g1_i1 encoding for a chloroplastic HS1 (heat stable protein 1) homologue of Arabidopsis, and c61165_g1_i1 with identity to AT5G02550, encoding for a small-sized hypothetical protein most likely localized in the nucleus, but with an unknown function. Interestingly, the analyses of the first 100 unigenes in 8Y/8W with Log2 [fold change (FC)] ≤ −5.3, reveals that 25% of unigenes encode for proteins involved in flavonoid and anthocyanin biosynthesis, including eight members of the MYB-related transcription factor family. These results are in agreement with the higher accumulation of flavonoids in the white parts of the tepals and the further accumulation of anthocyanin in the upper part of the tepals, which is tightly linked with flower development (Figs 1b and 2a). Therefore, we further analysed the expression levels of genes encoding for enzymes involved in flavonoid biosynthesis in the analysed tissues (Fig. S2), and observed that their expression levels correlated with the accumulation of flavonoids observed in the white sectors of S6 and S8.

### Identification of carotenogenic genes and expression analyses

**Figure 6:**
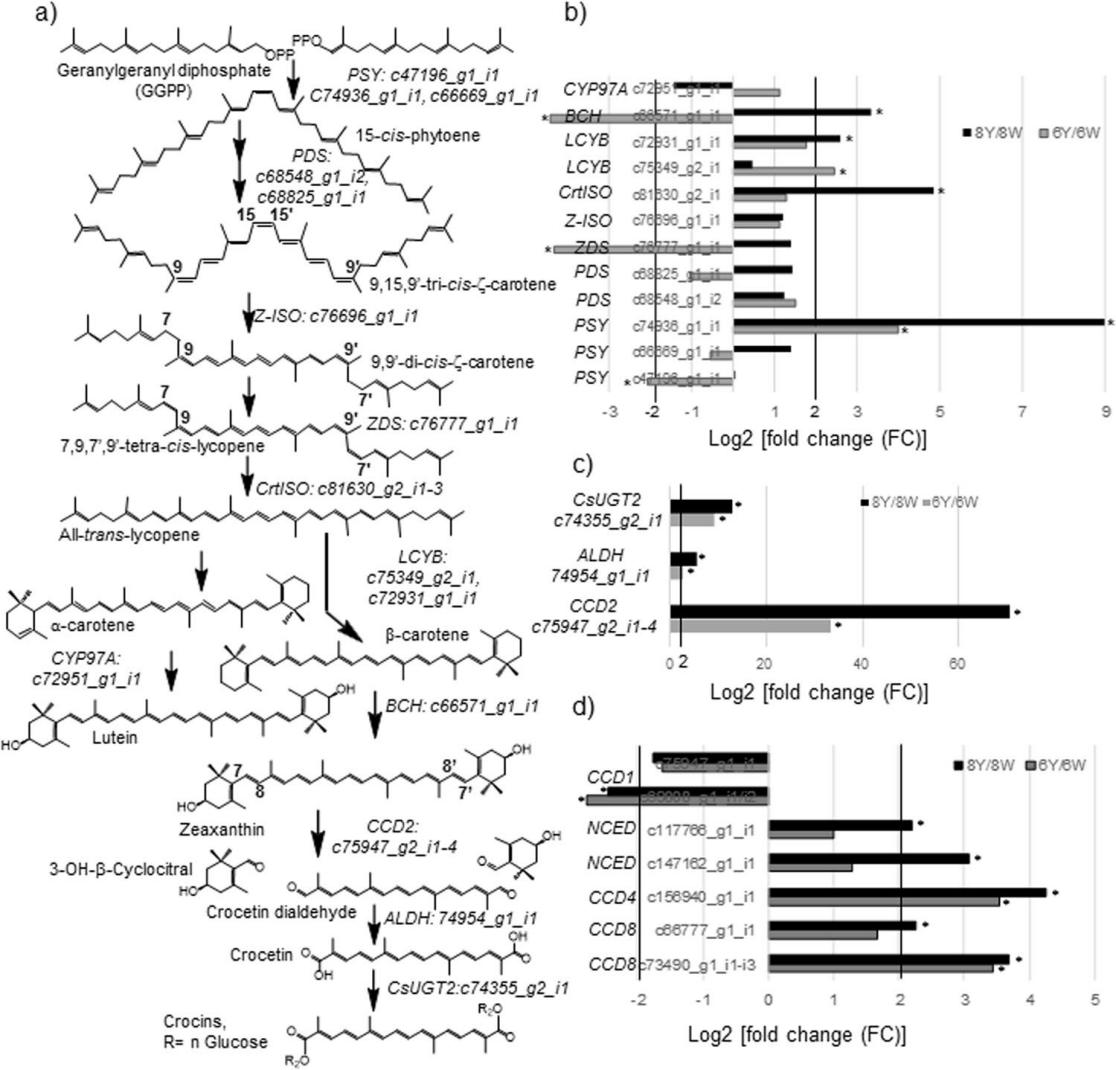
Expression levels of differentially expressed unigenes assigned to the carotenoid and apocarotenoid biosynthetic pathways in *C. sieberi*. (**a**) An overview of the carotenoid and crocin biosynthesis pathway enzymes and metabolites. Homologues for the different enzymes (in italics) were identified in the transcriptome assembly and unigenes codes are located beside each enzyme name. Abbreviations are as follows: PDS (phytoene synthase), PSY (phytoene synthase), PDS (phytoene desaturase), Z-ISO (15-*cis*-ζ-carotene isomerase), ZDS (Z-carotene desaturase), CrtISO (carotene isomerase), LCYB (lycopene-β-cyclase), BCH (β-carotene hydroxylase), CCD2 (carotenoid cleavage dioxygenase 2), ALDH (aldehyde dehydrogenase), CsUGT2 (*Crocus sativus* glucosyltransferase 2). (**b**) Differential expression analyses of carotenoid biosynthesis pathway genes identified in the pairwise comparisons of 6Y/6W and 8Y/8W. (**c**) Differential expression analyses of homologues to ALDH and UGT genes identified in the pair-wise comparisons of 6Y/6W and 8Y/8W. (**d**) Differential expression analyses of carotenoid cleavage dioxygenase genes homologues identified in the pair-wise comparisons of 6Y/6W and 8Y/8W.

Our differential expression analyses reveal the presence of *CCD*2 homologues in the top of transcripts differentially expressed in the 8Y/8W analyses (Table S1). We also searched for genes in the carotenogenic pathway and for genes specifically involved in apocarotenoid biosynthesis in saffron stigmas^5^. The first committed step in carotenoid biosynthesis in plants is catalysed by the enzyme phytoene synthase (PSY), which catalyses the conversion of geranylgeranyl pyrophosphate to phytoene (Fig. 6). Three genes (c47196_g1_i1; c74936_g1_i1 and c66669_g1_i1) with identity to *PSY* genes from *C. ancyrensis* tepals and stigma^23^ were identified in the *C. sieberi* transcriptomes. Only one of them, however, c74936_g1_i1 with identity to *CaPSY-II* from *C. ancyrensis*, was upregulated >3.9-fold in 6Y/6W and 8Y/8W (Fig. 6a). Interestingly, *CaPSY-II* is expressed at higher levels than *CaPSY-I* in flowers, and its expression followed the accumulation of crocins in the flowers of *C. ancyrensis*, suggesting its specific involvement in the accumulation of crocins^23^. The next steps are catalysed by a phytoene desaturase synthase (PDS) and ζ-carotene desaturase (ZDS), and two isomerases acting on ζ-carotene (Z-ISO) and poly *cis*-lycopene (CrtISO), resulting in the formation of lycopene^37^. Two genes, c68548_g1_i2 and c68825_g1_i1, with identities to *CaPDS-I* and *CaPDS-II* from *C. ancyrensis*, respectively^23^, were identified in the transcriptome, but none of them showed significant fold increase in the 6Y/8W and 8Y/8W transcriptomes (Fig. 6b). The same behaviour was observed for c76696_g1_i1, with identity to *Z-ISO* genes (Fig. 6a). Only one *ZDS* gene type, c76777_g1_i1, was identified in the analyses, and it showed significant downregulation in the 6Y/6W transcriptome (Fig. 6b). A *CtrISO* homologue, c81630_g2_i3, was identified in the transcriptomes. This transcript showed a 4.84-fold upregulation in the 8Y/8W (Fig. 6b). It has been suggested that CrtISO has a function controlling the production of apocarotenoids^15^, and this seems to be the case for crocetin accumulation in tepals and stigma in *C. ancyrensis*^23^, suggesting a similar role in *C. sieberi* tepals. Lycopene is further modified by lycopene cyclase enzymes (LCY), and in plants, there are two types of LCY (LCYB and LCYE). Two genes, c75349_g2_i1 and c72931_g1_i1, were identified with identity to *CaLCYB-I* and *CaLYCB-II* genes, respectively, from *C. ancyrensis*^23^ and saffron^38^, where CstLCYB-II is a chromoplast-specific lycopene β-cyclase and is one of the key enzymes controlling crocin accumulation in the stigma tissue of saffron^38^. C75349_g2_i1 was clearly upregulated in 6Y/8W, but not in 8Y/8W, while c72931_g1_i1, the *CstLCY-II* homologue, showed 2.59-fold upregulation in the 8Y/8W transcriptome (Fig. 6b). The LCYB enzymes introduce two β-ionone end groups on the lycopene molecule and produce β-carotene. In the extended carotenoid pathway, zeaxanthin is derived from β-carotene by the 3-hydroxylation of both β-ionone end groups. This reaction is catalysed by β-carotene hydroxylases^39^ (Fig. 6a). In *Crocus*, chromoplast-specific β-carotene hydroxylases involved in crocin biosynthesis have been previously identified^14^, and the transcript c66571_g1_i1 showed identity with the genes encoding for such specific enzymes. This transcript was downregulated in the 6Y/6W transcriptome, but showed a 3.35-fold upregulation in the 8Y/8W transcriptome (Fig. 6b). We further searched for α-carotene hydroxylases involved in lutein biosynthesis^39^ and found the transcript c72951_g1_i1, which did not show significant variation in the analysed transcriptomes (Fig. 6b).

In *Crocus* species, zeaxanthin is the precursor for the biosynthesis of crocetin dialdehyde in a reaction catalysed by the CCD2 enzyme. Transcripts with identity to *CCD*2 were identified in the transcriptome analyses (c75947_g2_i1–4) and showed the highest-fold induction in the yellow samples (Fig. 6c), with a 33.63-fold induction in the 6Y/6W transcriptome and a 70.7-fold induction in the 8Y/8W transcriptome. Crocetin dialdehyde is the substrate for aldehyde dehydrogenases to render crocetin. In the saffron transcriptome from stigma tissue, eight transcripts were identified encoding ALDH enzymes as potential candidates for crocetin dialdehyde dehydrogenase^27^. In addition, five *ALDHs* have been identified in the chromoplast proteome of red stigmas from saffron^21^. In the *C. sieberi* transcriptome, 12 transcripts showed identity to *ALDHs*, but only one (c74954_g1_i1) was upregulated in the yellow samples, suggesting its involvement in the biosynthesis of crocetin in these samples (Fig. 6c). Crocetin is further modified by glucosyltransferase enzymes^20^. The transcript c74355_g2_i1 showed 91% identity with the gene encoding for GtCs2, involved in crocetin glucosylation^20^. This transcript (named CsUGT2) showed a 9.33 and 13.02-fold increased expression in 6Y/6W and 8Y/8W transcriptomes, respectively (Fig. 6c).

Other carotenoid cleavage dioxygenases, previously described in saffron^40–43^ were also identified in the transcriptome. Homologues to other CCDs involved in volatiles (VOCs; CCD1/4), strigolactones (SL; CCD7/8) and abscisic acid (ABA; 9-*cis*-epoxycarotenoid dioxygenase (NCED)) were also found in the *C. sieberi* transcriptome. They included *CsCCD1* (unigenes c69808_g1_i1/i2; c75947_g1_i1), *CsCCD4a/b* (unigene c156940_g1_i1), *CsCCD7* (c127590_g1_i1), *CsCCD8a* (unigenes c73490_g1_i1/i2/i3) and *CsCCD8b* (c66777_g1_i1). *NCED* (c147162_g1_i1 and c117766_g1_i1) was also detected in all the samples and showed different expression levels (Fig. 6d). Interestingly, the *CCD1* homologues were the only ones downregulated in the yellow tissues and upregulated in the 8W tissue, while the other *CCDs* transcripts showed a significant fold induction in the 8Y sample (Fig. 6d). The expression of *CCD1* in the white samples could be related to the higher concentration of the apocarotenoid 3-hydroxy-β-ionone in the 8W sample (Fig. 2d).

We also look for homologues of the *Orange* gene (*Or*), originally discovered in cauliflower (*Brassica oleracea*). The *Or* gene enhances sink strength by triggering the biogenesis of chromoplasts in non-green tissues^44^. For the identified unigenes c74014_g1_i1–2, however, no significant differences were found in the 6Y/8W (2.35), 6W/8W (2.12) and 8Y/8W (2.56) transcriptomes.

**Figure 7:**
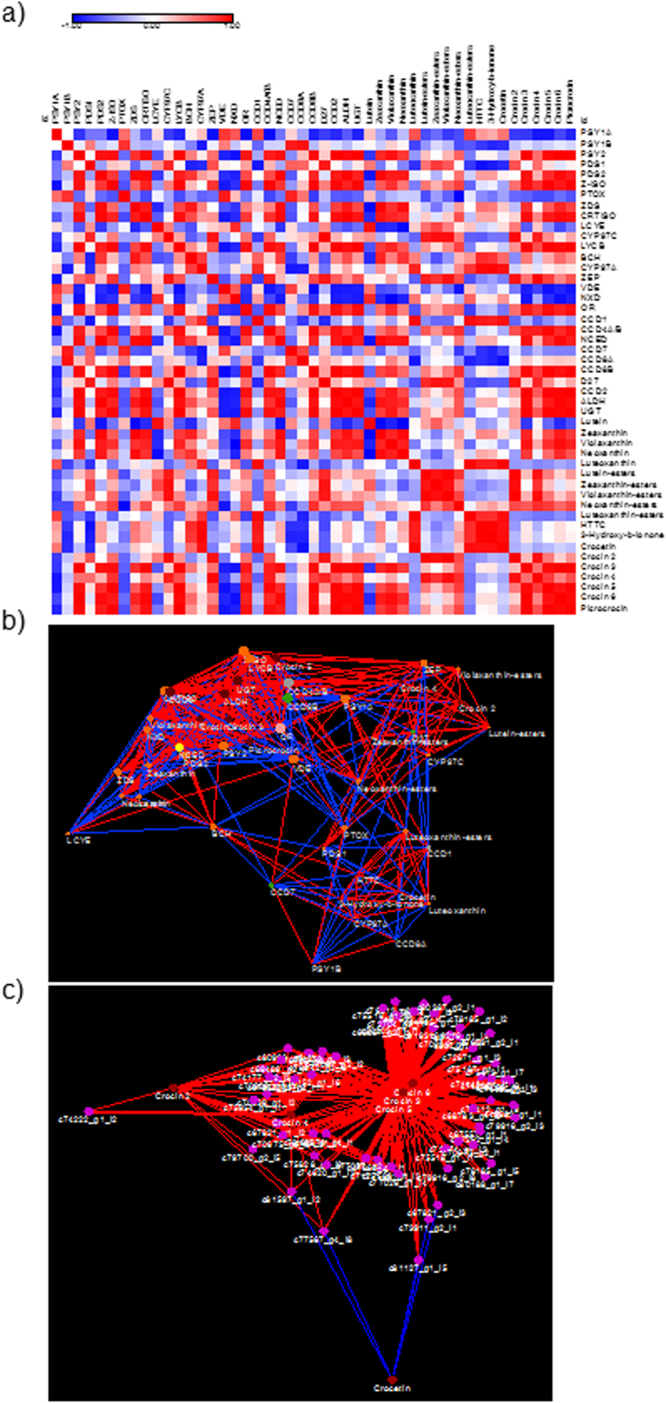
Metabolite-gene correlation analyses associated with carotenoid/apocarotenoid metabolism. (**a**) Heatmap plots for gene-metabolite relationships in carotenoid/apocarotenoid biosynthesis. Legend on the right indicates the corresponding names of genes and metabolites. Red and blue shaded boxes indicate, respectively, different extents of positive and negative correlations; white boxes indicate no correlation. (**b**) Carotenoid/apocarotenoid transcript/metabolite correlation network using a prefuse force-directed layout (only r > 0.65). Different colors identify genes/metabolites involved in the synthesis of carotenoids (orange), crocins (burgundy), VOCs (gray), SLs (green) and ABA (yellow), while Or, a carotenoid pathway regulator, is indicated in pink. Blue and red edges refer, respectively, to negative and positive correlations; only correlations >0.65 are shown. (**c**) Transcription factor transcripts/crocins correlation network using a prefuse force-directed layout (only r > 0.65). Transcription factors are indicated in pink and crocins in brown. Red edges refer to a positive correlation. Networks are visualized as circles with nodes of different sizes according the node strength (ns). Lines joining the nodes represent positive (red) and negative (blue) correlations, of width proportional to each corresponding |ρ|.

Hierarchical Clustering (HCL) of carotenoid and apocarotenoid genes (Fig. 7a) allowed highlighting transcripts most strongly associated with crocin biosynthetic genes: thus, *CCD*2 was grouped in a cluster including *PSY*2, the key gene of the *Crocus* carotenoid pathway; *LCYB*, responsible for lycopene cyclization; the tentative *UGT* for crocetin glucosylation; and *CCD8B*, involved in strigolactone synthesis. Interestingly, two other *PSY* and *CCD8* isoforms, *PSY1B* and *CCD8A*, were found to be tightly associated. To gain a more comprehensive knowledge about the *C. sieberi* carotenoid pathway, we integrated transcript and metabolite data using the Pearson correlation coefficients (ρs). First, symmetric correlation matrix visualization (Fig. 7a) was exploited to show the most robust relationships within carotenoid and apocarotenoid biosynthesis: notably, all crocins were strongly and positively correlated with several early (*PSY2*, *PDS2*, *Z-ISO*, *ZDS*, *CRTISO*) and late (*CYP97C*, *LCYB*, *BCH*, *ZEP*) genes as well β,β-xanthophylls in free (zeaxanthin, violaxanthin, neoxanthin) or esterified forms. Finally, correlation coefficients were also used to build a correlation network with a prefuse force-directed layout (Fig. 7b), which generates an *ad hoc* topology in relation to the dataset under study. In agreement with the previous findings, this strategy evidenced an area of high correlative power with a positive sign, which comprises a series of nodes (transcripts and metabolites) of carotenoid (*Z-ISO*, *ZDS*, and *CRTISO*) and crocin metabolism (Crocin 2–5, *CCD2*, *ALDH*, *UGT*, etc.). In addition, such an approach can be used to unravel negative hubs (e.g., nodes yielding a high number of negative correlations), which could potentially represent elements contrasting crocin accumulation; this group included well-known genes and metabolites opposing to β,β-branch carotenoids (as zeaxanthin, the crocins precursor), such as *LCYE* and lutein. Furthermore, additional transcripts of interest were represented by *PSY1A/B*, *PDS1*, *P-TOX*, and *VDE*.

### Genes participating in chromoplast biogenesis

Chromoplast differentiation from nonphotosynthetic plastids occurs frequently in a number of plant tissues, such as carrot root, sweet potato tubers, and watermelon, mango, and citrus flesh^6^. We searched for those upregulated transcripts with a plastid location in the yellow samples, encoding for proteins involved in a specific functional class (Fig. S4). We found 520 unigenes encoding for proteins most likely located in plastids, with different expression levels in the analysed transcriptomes (Fig. S4a). Unigenes with a Log2 [fold change (FC)] ≥ 2 in 8Y/8W represent 24.42%, in 6Y/8W represent 22.88% and in 6W/8W represent 21.92%. These unigenes were categorized into functional classes based on the protein products (Fig. S4b). The analysed unigenes mainly fell into five categories: redox and stress, transport, lipid metabolism, photosynthesis and metal handling. The upregulated unigenes in 6Y/8W and 8Y/8W were mostly present in transport, lipid metabolism, metal handling, cofactor and vitamin metabolism and in secondary metabolism related to carotenoids (Fig. S4b). With regard to the transport class, secondary active sulfate transmembrane transporter activity is clearly down regulated in the yellow samples; in the redox and stress class, unigenes encoding for NCED enzymes showed a Log2 [fold change (FC)] > 2 only in the yellow samples. A number of unigenes encoding for proteins involved in photosystems PSI and PSII, in light reactions and in photorespiration were detected (Table S2) but were mostly upregulated in the white samples. Unigenes encoding for proteins involved in the starch biosynthesis pathway were also detected, including starch synthase, ADP-glucose pyrophosphorylase, glucose-1-phosphate adenylyltransferase, and 1,4-α-glucan branching protein. In addition, unigenes encoding for proteins involved in starch degradation, such as β-amylase, glucan phosphorylase and water dikinase, were also detected (Table S3). Interestingly, the unigenes associated with starch metabolism were downregulated in 6Y/8W and 8Y/8W, suggesting a dynamic starch turnover in the 8W stage. Notably, sugar transporters were upregulated in 8Y/8W. In most plants, glucose 6-phosphate is the preferred hexose phosphate taken up by non-green plastids. The transporter responsible for this import into the plastids is known as the glucose 6-phosphate transporter, and a transcript similar to genes encoding this transporter was seen to be enriched in 8Y/8W, indicating that the reducing power of the plastids in the yellow samples may be provided by the light-independent production of NADPH through the glucose-6-phosphate dehydrogenase activity present in the analysed samples (Table S3).

### Analyses of transcription factors associated with apocarotenoid biosynthesis

Although the cloning and functional characterization of the enzymes involved in carotenoid biosynthesis has been quite successful in recent decades, knowledge about the regulation of the carotenoid and apocarotenoid pathways is very limited. Studies of many plants, including saffron and other *Crocus* species, show a close association between carotenoid accumulation and transcript abundance for many of the carotenogenic genes, suggesting that carotenoid and apocarotenoid biosynthesis is primarily influenced at the transcriptional level^14,23,38^. A total of 65 transcriptional factors were upregulated in the yellow samples, and positively correlated to the crocins content (Fig. 7c). Table 5 shows the top 20 transcription factors that were significantly more abundant in yellow tissues, and whose levels increased with the developmental stage as observed for the carotenogenic and apocarotenogenic transcripts. Transcription factors that belong to the *ERF/AP2* (APETALA2/ethylene response factor), *AP2/EREBP* (APETALA2/ethylene-responsive element-binding protein), *Dof-C*_*2*_*H*_*2*_ (DNA binding with one finger), and *NAC* (NAM, ATAF1–2, CUC2) were present among those with a clear differential expression in yellow samples. The *ERF/AP2* represents a superfamily that plays an important role in different processes including growth and development^45^, and biosynthesis of secondary metabolites^46–50^. A member of this family of transcriptional factors, AtRAP2.2, binds to the ATCTA *cis* acting element present in the Arabidopsis *PSY* and *PDS* genes promoters^51,52^. Further, an allelic variant of the *Or* gene from *Cucumis melo* (*CmOr*) is associated with differentially expressed genes from the *AP2* family^53^. The *AP2/EREBP* is a large family of transcription factors genes that play a major role in controlling developmental processes. Furthermore, members of the *AP2/EREBP* family are implicated in the integration of signals derived from organelles in retrograde feedback loops and in stress acclimation^54^. The *Dof-C*_*2*_*H*_*2*_ family binds specifically to DNA sequences with a 5′-(A/T)AAAG-3′ core, and also plays diverse roles in plant growth and development^55^, and recently one member of this family has been shown to be potentially involved in the transcriptional regulation of β-carotene accumulation in carrot^56^. The *NAC* domain does not possess any known DNA-binding motif, but one face of the domain is rich in positive charges and is probably involved in binding DNA^57^. The complex regulation of *NAC* transcription factors includes microRNA (miRNA)-mediated cleavage of mRNAs and ubiquitin dependent proteolysis. These factors also play roles in the regulation of the expression of flavonoid biosynthesis-related genes, ABA signalling, senescence, and cell division^58^. The expression patterns of these top twenty transcription factors that were differentially expressed in the yellow samples were examined by qRT-PCR, and the results were consistent with the results from the transcriptome analysis (Fig. S5). From the results, we found that c81876_g1_i1 (ARF transcription family) and c47864_g1_i1 (MYB transcription family) contigs showed the highest expression levels in the yellow sectors, while the contigs c45682_g1_i1 and c82083_gi_i2, both from the ERF/AP2 transcription factor family, were the ones with the lower expression levels in the yellow sectors of both developmental stages (Fig. S5).

**Table 5:** Transcription factors differentially expressed in the analysed samples with Log2 [fold change (FC)] ≥2.

Contig	6Y/8W	6W/8W	8Y/8W	Description	Trancription factor family
c61165_g1_i1	8,006286	1,648608	12,536966	TAIR|locus:2181793 - symbol:AT5G02550	—
c75020_g1_i1	9,265837	1,163854	9,443675	MADS–box transcription factor AAS67611.1.	MADS-MIKC
c47864_g1_i1	5,844103	1,489022	6,973803	TAIR|locus:2163766 - symbol:TRY AT5G53200	MYB
c19477_g1_i1	5,51078	1,233083	5,602283	TAIR|locus:2156529 - symbol:LATE AT5G48890.1	C2H2
c76152_g1_i6	3,889788	1,252883	4,376977	TAIR|locus:2055007 - symbol: AT2G44940 DREB subfamily	AP2-EREBP
c70036_g1_i1	3,595363	1,03479	4,563596	TAIR|locus:2184133 - symbol: AT5G10150	FLC
c81876_g1_i1	3,529221	−1,388653	3,913438	TAIR|locus:2152642 - symbol:ARF8 AT5G37020	ARF
c61931_g1_i1	3,2107	−1,050902	6,123365	TAIR|locus:2016344 - symbol:OFP14 AT1G79960	OFP
c78185_g1_i2	3,077502	1,988864	3,866639	TAIR|locus:2041105 - symbol:SGR5 AT2G01940	C2H2
c77057_g3_i1	3,033889	1,271778	3,631152	TAIR|locus:2047246 - symbol:COL3 AT2G24790	Orphans
c72262_g1_i4	3,009898	1,004396	5,106027	UNIPROTKB|Q7XDD0 - symbol:G1L5 “Protein G1-like5”	NAC
c79916_g2_i6	2,834383	1,666357	3,738728	UNIPROTKB|A2XA73 - symbol:GRF1 “Growth-regulating factor 1”	GRF
c67557_g1_i2	2,734267	1,070491	3,770351	TAIR|locus:2056266 - symbol:LOV1 AT2G02450	NAC
c307_g1_i1	2,452616	1,041774	6,332279	UNIPROTKB|Q75G11-symbol:OSJNBa0013A09.16	BHBL
c66216_g1_i1	2,433348	−1,38425	3,7125	TAIR|locus:2201103 - symbol:DREB26 AT1G21910	AP2-EREBP
c50202_g1_i1	2,302822	1,640719	4,777601	TAIR|locus:2010587 - symbol: AT1G04880	ARID/BRIGHT
C82083_g1_i1	2,270527	1,640057	2,07305	TAIR|locus:2154272 - symbol: CRF3 AT5G53290	ERF/AP2
c45682_g1_i1	2,112074	1,543998	2,628103	TAIR|locus:2032510 - symbol:ERF12 AT1G28360	ERF/AP2
c79700_g2_i5	2,057499	1,430061	2,119337	TAIR|locus:2085859 - symbol:AT3G18960	AP2/B3
c67821_g2_i3	2,033657	1,701241	2,54532	TAIR|locus:2202795 - symbol:AtHB23 AT1G26960	HB

## Conclusions

The comparative transcriptome of *C. sieberi* tepal sectors in two developmental stages provide a global landscape of differentially expressed genes in the same tissue with contrasted secondary metabolite accumulation. The transcriptomes’ sequence data allowed the identification and characterization of the expression levels of transcript encoding key enzymes involved in flavonoid and apocarotenoid metabolic pathways, providing insight into the biosynthesis of these secondary metabolites in *C. sieberi*. The comparative gene expression levels in relation to the flavonoid and apocarotenoid contents in the analysed tissues revealed the complexity of gene expression and metabolite accumulation in the crocin biosynthesis pathway. Further, Raman spectroscopy has enabled the unambiguous subcellular location of crocetin and crocins, which were never shown before in Crocus.

## Materials and Methods

### Chemicals and plant materials

Chemicals and reagents were obtained from Sigma-Aldrich unless otherwise stated. Tepals and stigma were obtained from *C. sieberi* grown under field conditions in the Botanical Garden of Castilla-La Mancha (Albacete, Spain), collected at different developmental stages, dissected and frozen in liquid nitrogen and stored at −80 °C until required.

### Extraction and analysis of apocarotenoids by HPLC-DAD

Dissected samples from tepals at different developmental stages were ground in liquid nitrogen with the mixer mill MM400 (Retsch GmbH, Haan, Germany) in a 1.5 ml Eppendorf tube, and then extracted with 1 ml Tris-HCl (50 mM, pH 7.5) (containing 1 M NaCl) and incubated for 10 min on ice. 1 ml of CHCl_3_ was then added, mixed, and the extract incubated on ice for an additional 10 min followed by centrifugation at 3,000 g for 5 min at 4 °C. The lower CHCl_3_ phase was evaporated under N_2_ gas and the dried residues were stored together with the upper aqueous phases at −80 °C until analysis by HPLC-DAD. All the assays were performed in triplicate.

The HPLC methods used for the analysis and detection of glycosylated apocarotenoids have been previously described^22,23^.

### Extraction and analysis of apocarotenoids by HPLC-DAD-HRMS

Extraction and HPLC separation of carotenoids and apocarotenoids/phenylpropanoids was performed as previously described^59–61^. High-resolution mass spectrometry (HRMS) analysis was carried out using a Q-Exactive system (ThermoFisher Scientific), operating in both positive and negative ion modes, and in the mass range 110–1600 m/z. The ionization of flavonoids and apocarotenoids was performed using a heated electrospray ionization (HESI) source with nitrogen as sheath (40 units) and auxiliary (35 units) gas, respectively. Vaporizer and capillary temperatures were set at 250 and 240 °C, respectively; discharge current was 4.5 μA, while S-lens RF level was 60. The ionization of carotenoids was carried out using an atmospheric-pressure chemical ionization (APCI) source. Nitrogen sheath and auxiliary gas were used at, respectively, 30 and 20 units. The vaporizer and capillary temperatures were set at 270 and 250 °C, respectively, while the discharge current and S-lens RF levels were 5.0 μA and 50, respectively. Metabolite identification was performed as previously described^62^. Carotenoids and crocins were quantified in an absolute way as previously described^63^, while phenylpropanoids were relatively quantified as previously reported^60^.

### Hyperspectral confocal Raman micro-spectroscopy (RS measurements)

For RS analysis, tepals were displaced in a quartz slide humidified with a drop of water.

The Raman system used was an inVia Renishaw (Apply Innovation, Gloucestershire, UK), which comprises a 532 nm laser that supplies an excitation beam of approximately 10 mW power that is focused onto the sample *via* a microscope with 50x objective and using a backscattered configuration. The Raman spectrum is recorded on a deep depletion charge-coupled device (CCD) detector (Renishaw RenCam). The recorded Raman spectrum is digitalized and displayed on a personal computer using Renishaw WiRE software, which allows the experimental parameters to be set. Cell images of areas approximately 40 × 20 µm were measured with a pixel resolution of 1 µm. The acquisition time for each pixel was 1 s. Multivariate methods were used to analyse Raman images by using Matlab and PLS Toolbox.

### Transmission electron microscopy (TEM)

Fresh tepals were dissected in small portions and fixed overnight by immersion in 2% paraformaldehyde and 2% glutaraldehyde in 0.1 M phosphate buffer (PB, pH 7.4) at 4 °C. Subsequently, samples were washed in PB and postfixed in 1% osmium tetroxide for 30 minutes in darkness. After several washes in PB, samples were treated with 0.1% uranyl acetate for 30 minutes in darkness. Then, samples were dehydrated in graded ethanol series, propylene oxide and embedded in epoxy resin (Durcupan). Polymerisation was performed at 60 °C for 48 h. Ultrathin sections (70 nm thickness) were cut on an ultramicrotome (Reichert Ultracut E; Leica, Austria) and collected on 200—mesh copper grids. Ultrastructural analyses were carried out on a Jeol-1010 Transmission Electron Microscope (Jeol Ltd., Akishima, Japan).

### Samples RNA extraction for sequencing

Total RNA was extracted from dissected white and yellow tepal sectors in two different developmental stages using TRIzol reagent (Invitrogen, Carlsbad, USA). An average of 10–15 μg total RNA from each sample was sent to Macrogen Inc. (Seoul, South Korea; www.macrogen.com) for library construction and sequencing. The four libraries were constructed using the TruSeq RNA Sample Prep kit (Illumina, San Diego, USA) and were sequenced using a HiSeq. 2000 sequencer (Illumina) to generate inward paired-end reads of 100 bp.

### Data processing, transcriptomes assembly and annotation

The absence of a reference genome for *C. sieberi*, together with the high coverage of the four sequenced RNA libraries, led to the use of a *de novo* transcriptome assembly pipeline. At first, raw RNA-seq data were trimmed from adaptors and sequencing artefacts, as well as low quality fragments, using the NGS QC Toolkit. The high-quality reads obtained were then subjected to *in silico* normalization prior to *de novo* assembly to reduce the sequencing coverage of highly represented regions with a fragment density higher than 30×. In that way, computational complexity is reduced without affecting the quality of the assembled transcriptome. Using the *de novo* transcriptome assembler tool from Trinity^64^, the normalized data were assembled with a minimum fragment overlap of 40 bp. Only unigenes longer than 300 bp were included in the assembled *C. sieberi* transcriptome and subjected to further analyses, including gene ontology (GO) sequence annotation using Blast2Go^65^.

### Analysis of differentially expressed transcripts in yellow and white samples from *C. sieberi* tepals

Using the assembled transcriptome as a reference for *C. sieberi*, the sequenced filtered libraries were subjected to transcriptome expression analysis. The libraries were mapped to the reference transcriptome using Bowtie (http://bowtie-bio.sourceforge.net/) with default parameters^66^. The abundance of aligned reads was estimated by Cufflinks v.2.1.1 (http://cole-trapnell-lab.github.io/cufflinks/), which accepted aligned reads and assembled the alignments into a clear, simple set of transcripts. Next, RNA-seq fragment counts were measured by the unit of fragments per kilobase of exon per million fragments mapped (FPKM). A gene was considered low expressed if the FPKM value was ≤2, moderately expressed if the FPKM value was >2 and ≤10, and highly expressed if the FPKM value was >10. The determination of the 10 most abundant transcripts was performed by mutual comparison among the 6Y transcripts, 6W transcripts, 8Y transcripts, and 8W transcripts, the genes that existed in all of the five tissues were defined as common genes, and was possible to evaluate their expression levels by their FPKM values. For differential expression analysis, the values of log2 (FPKM + 1) were calculated, and these were normalized by quantile normalization. P-values were obtained by t-test between each sample, and fold changes were calculated with the mean log2 (FPKM + 1) values, gene by gene. All data analysis of DEG was conducted using R 2.14.1 (http://www.r-project.org). To perform this analysis, we included the R Bioconductor package DESeq in our pipeline^36^. Differential expression between developmental stages was screened by detecting genes with statistical significance. The count matrix for all sequenced samples was also used to calculate a Euclidian distance matrix, which was used for hierarchical sample clustering. According to the most similar transcriptome profile calculated by a single linkage method, a dendrogram and a heatmap were generated, correlating sample expression profiles into colours ranging from red (identical profiles) to green (the most different profiles) of 6W vs 8W, 6Y vs 8W and 8Y vs 8W. The three database annotations were used to identify genes involved in the flavonoid and carotenoid pathway, as well as the transcription factors. The identified candidate transcription factors were validated in PlantTFcat (http://plantgrn.noble.org/PlantTFcat/).

### RT-qPCR analysis

For qRT‐PCR analysis, total RNA (2 μg) from yellow and white parts of tepals in developmental stages S6 and S8 was treated with RQ1 DNase (Promega) and reverse‐transcribed using oligo dT primers and a first-strand cDNA synthesis kit (GE Healthcare Life Sciences, Buckinghamshire, UK) according to manufacturer’s instructions. PCRs were carried out in triplicate using 10 ng of template cDNA, 200 nM target‐specific primers (Table S4) and LightCycler 480 SYBR Green I Master (Roche) in a StepOne™ Thermal Cycler (Applied Biosystems, Foster City, California, USA) in a volume of 10 μl, and analysed using StepOne software v2.0 (Applied Biosystems, Foster City, California, USA). The relative expression levels for each gene were normalized to the expression level of 18 S rRNA as described previously {Ahrazem, 2016 #3211}.

### Data integration

Hierarchical clustering (HCL), correlation matrix and networks were performed as previously described^59,67,68^, with slight modifications: in order to link genes and metabolites, we normalized all the data to the 6W stage; subsequently, once obtained adimensional values, we applied the Pearson correlation coefficient to any data pair (gene-gene, metabolite-metabolite or gene-metabolite), which were then utilized to build a symmetric matrix or networks. Different shades of blue and red highlighted, respectively, negative and red correlations, and values equal or above |0.65| were considered statistically significant. In the network analyses force-directed layout was used, as well as visualization using crocins as central hubs and building edge length inversely proportional to the Pearson correlation coefficient (|p|). In the network diagrams, the edge thickness is proportional to the absolute value of the Pearson correlation coefficient (|ρ|), whereas node sizes are proportional to their node strengths. Direct (ρ > 0) and inverse (ρ < 0) correlations are shown in red and blue, respectively. In order to distinguish transcripts from crocins, different node shapes were used. Networks were visualized as circle layouts with Cytoscape version 2.6.2 (www.cytoscape.org).

### Data availability

The raw Illumina data generated in this study were deposited in the NCBI Sequence Read Archive (SRA) under the BioProject accession number PRJNA413953.

## Electronic supplementary material

Supplementary Information
