# Supplementary material for: Transcriptome analysis in tissue sectors with contrasting crocins accumulation provides novel insights into apocarotenoid biosynthesis and regulation during chromoplast biogenesis
Source: Sci Rep. 2018 Feb 12;8:2843. doi: 10.1038/s41598-018-21225-z (PMC5809551; doi:10.1038/s41598-018-21225-z)
Supplement: Supplementary file 1 — Supplementary Information [file 41598_2018_21225_MOESM1_ESM.pdf]

Transcriptome analysis in tissue sectors with contrasting crocins accumulation provides novel insights into apocarotenoid biosynthesis and regulation during chromoplast biogenesis

Oussama Ahrazem[1,2], Javier Argandoña[1], Alessia Fiore[3], Carolina Aguado[4], Rafael Luján[4], Ángela Rubio-Moraga[1], Mónica Marro[5], Cuauhtémoc Araujo-Andrade[5], Pablo Loza-Alvarez[5], Gianfranco Diretto[3], Lourdes Gómez-Gómez[1,]*

[1]Instituto Botánico, Departamento de Ciencia y Tecnología Agroforestal y Genética, Facultad de Farmacia, Universidad de Castilla-La Mancha, Campus Universitario s/n, 02071 Albacete, Spain

[2]Facultad de Ciencias Ambientales y Bioquímica, Universidad de Castilla-La Mancha, Campus Tecnológico de la Fábrica de Armas, Av. Carlos III s/n, 45071 Toledo, Spain

[3]Italian National Agency for New Technologies, Energy, and Sustainable Development, Casaccia Research Centre, 00123 Rome, Italy

[4]Synaptic Structure Laboratory, Instituto de Investigación en Discapacidades Neurológicas (IDINE), Departamento Ciencias Médicas, Facultad de Medicina, Universidad Castilla-La Mancha, Campus Universitario s/n, 02071 Albacete, Spain

[5]ICFO-Institut de Ciencies Fotoniques, The Barcelona Institute of Science and Technology, Av. Carl Friedrich Gauss 3, 08860 Castelldefels, Spain

*Correspondence: Lourdes Gómez-Gómez (Marialourdes.gomez@uclm.es)

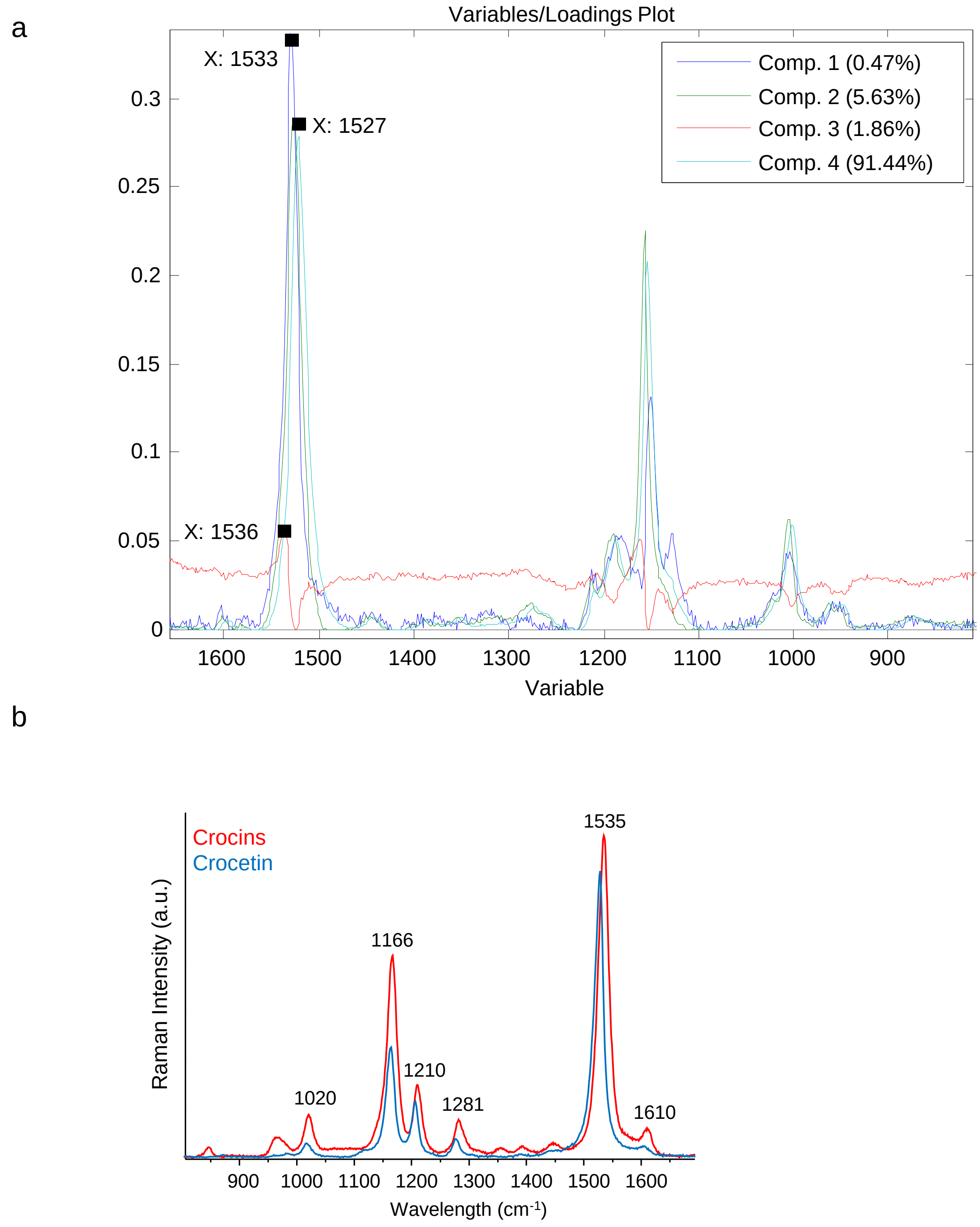

Figure S1. Raman spectroscopic features and absorbance spectra of the *C. sieberi* pigments. (a) Raman spectra of the compounds present in *C. sieberi* tepals using an excitation wavelength of 532 nm. (b) Fingerprint region of Raman spectra of purified crocetin and crocins from *C. sieberi* tepals.

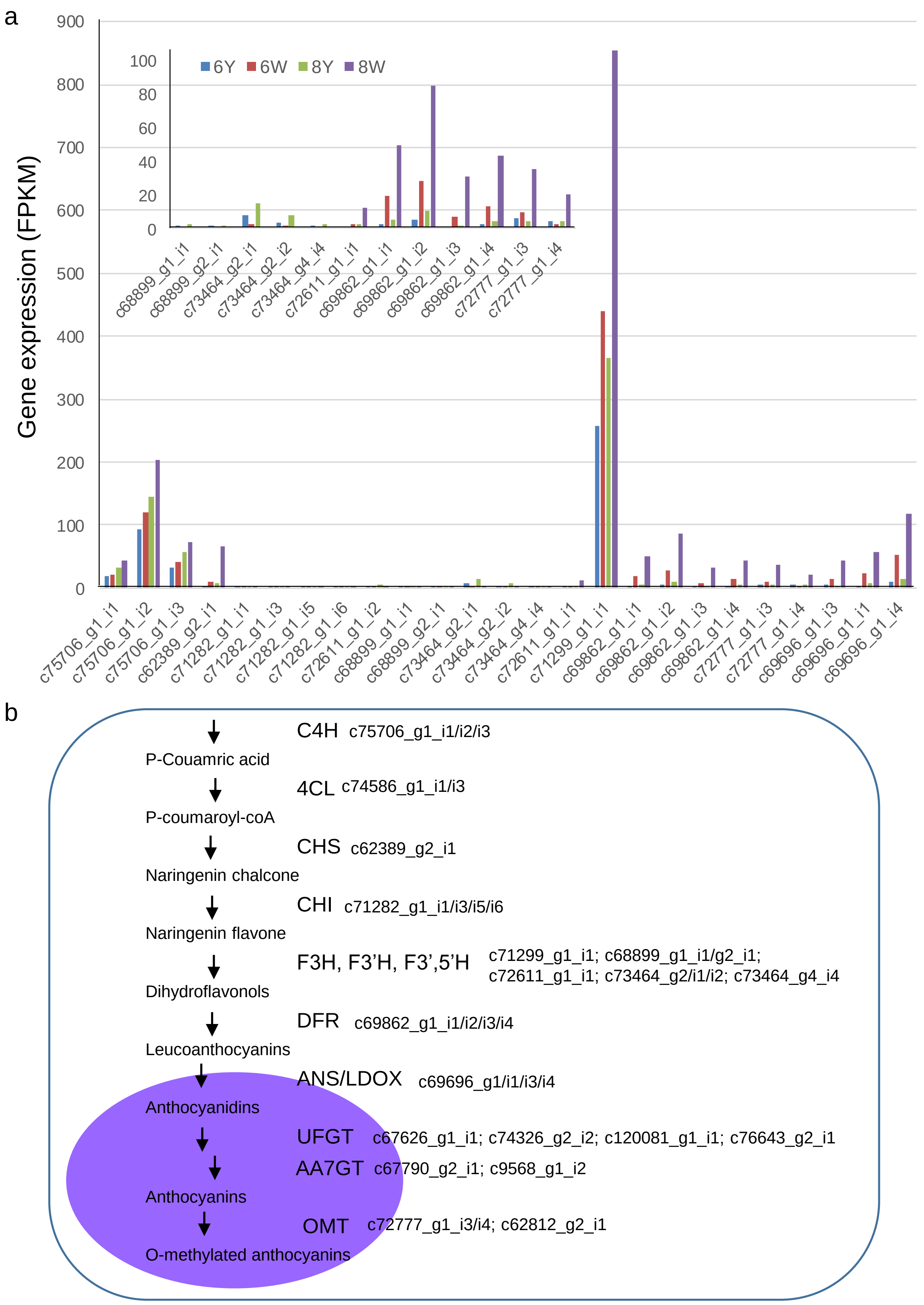

Figure S2. Flavonoid biosynthesis pathway and *C. sieberi* flavonoid unigene expression in tepal sectors. (a) Expression profiles of unigenes with homology to genes enconding enzymes involved in flavonoid biosynthesis. Unigene IDs are shown in Supplementary Table S5. (b) An overview of the flavonoid biosynthesis pathway enzymes and metabolites. Abbreviations are as follows: C4H (cinnamate-4-hydroxylase), 4CL (4-coumarate:CoA ligase), CHS (chalcone synthase), CHI (chalcone isomerase), F3H (naringenin 3-dioxygenase), FNS (flavone synthase), DFR (dihydroflavonol 4-reductase), LDOX (leucoanthocyanidin dioxygenase), F3′H (flavonoid 3′-monooxygenase), F3′5′H (flavonoid 3′,5′-hydroxylase), FLS (flavonol synthase), UFGT (UDP glucose flavonoid 3-o-glucosyl transferase), AA7GT (anthocyanin 7-O-glucosyltransferase) and OMT (anthocyanidin O-methyl transferase).

a

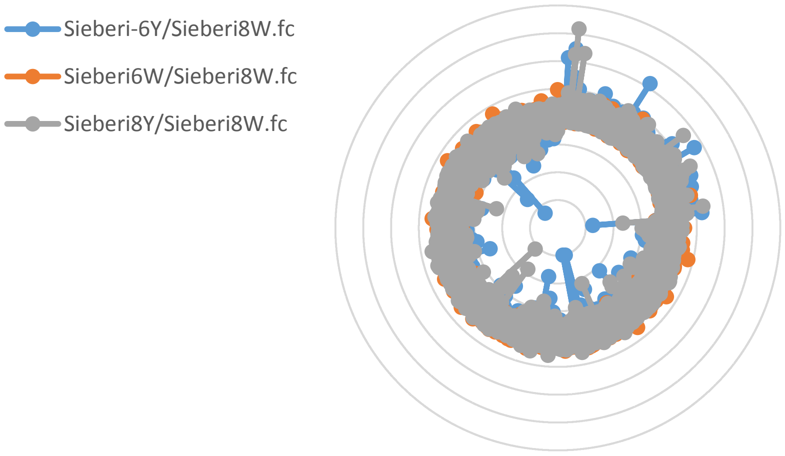

b

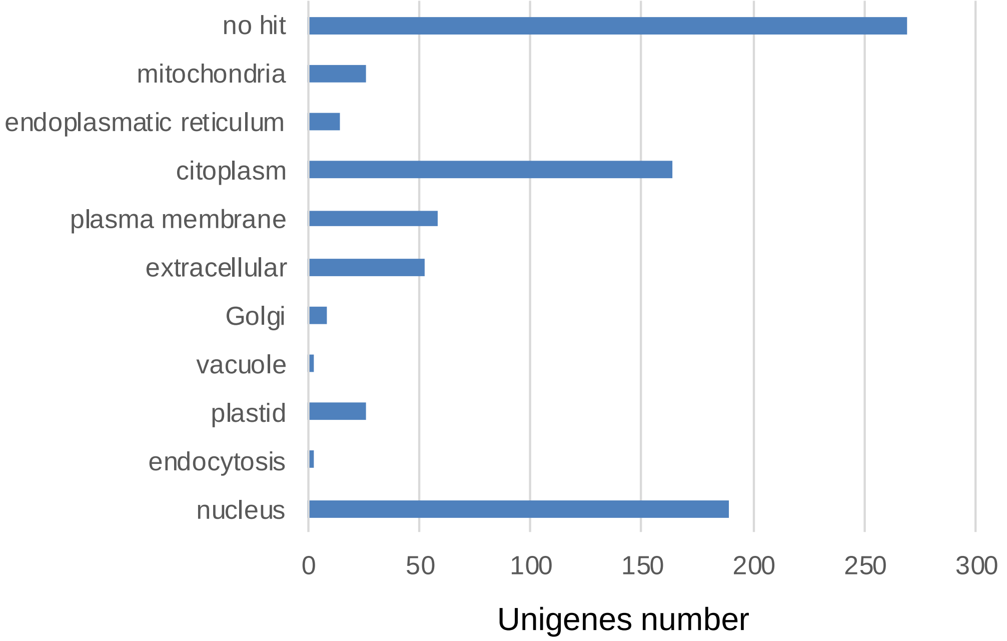

Figure S3. Upregulated genes in the yellow tepal samples of *C. sieberi*. (a) Diagram showing the presence of exclusively up-regulated unigenes in 6Y/8W and 8Y/8W transcriptomes. (b) Biological functions of positively selected genes in pair-wise comparisons among 6Y/8W and 8Y/8W.

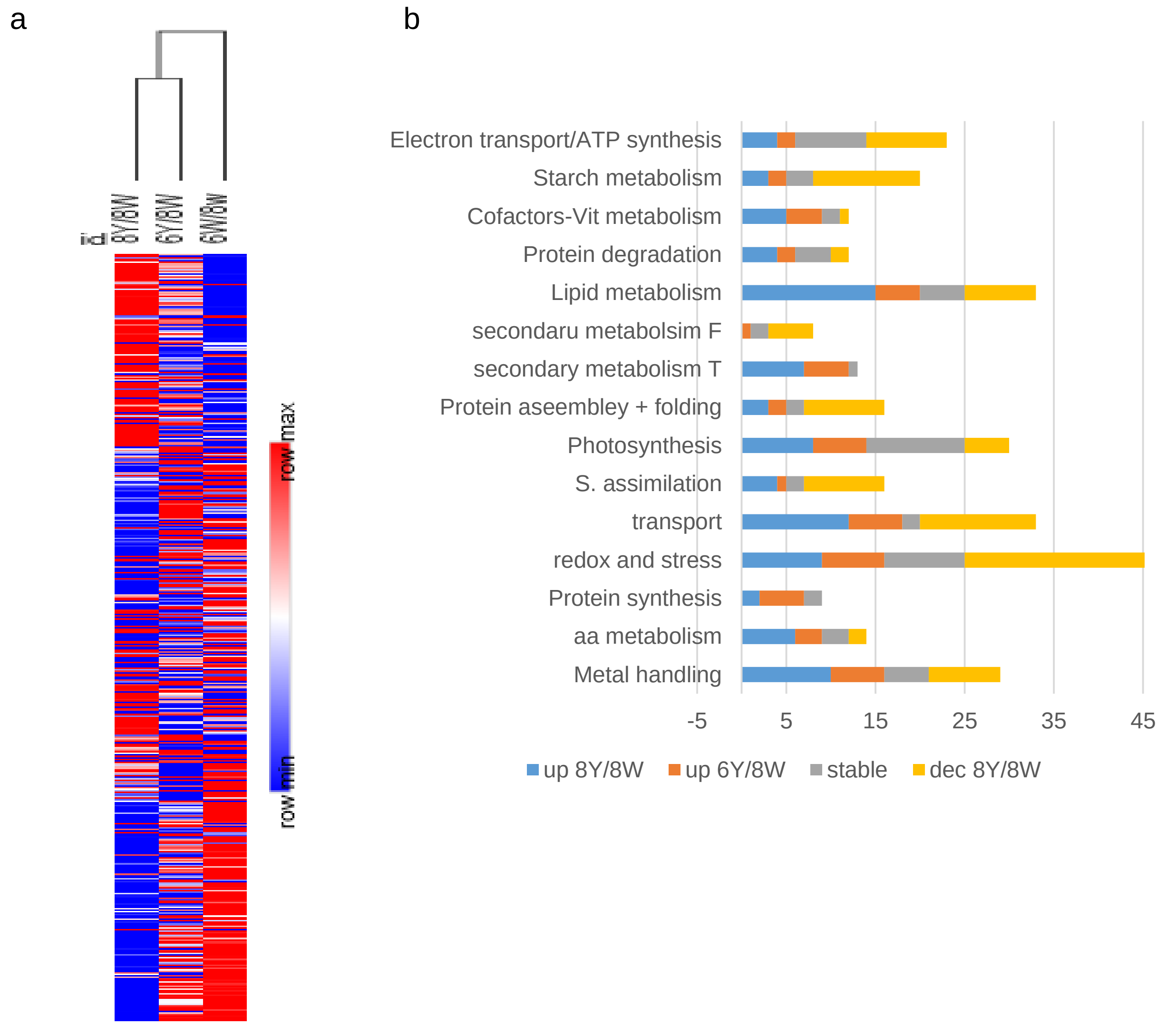

Figure S4. Analysis of plastid genes differentially expressed. (a) Heatmap comparing significant differentially expressed contigs associated to plastid genes. Intensity of color indicates expression levels. (b) Unigenes categorized into functional classes based on their protein products.

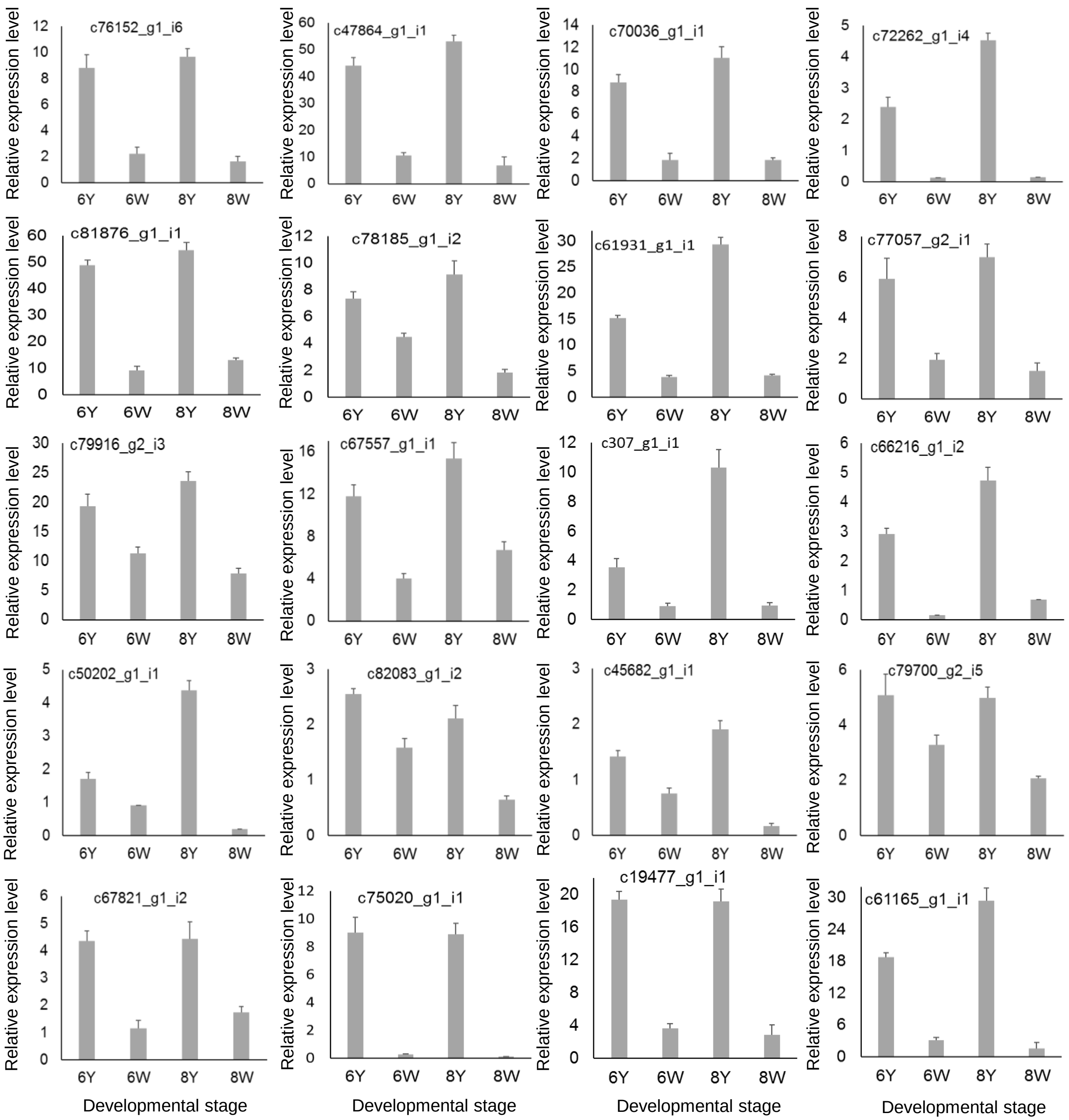

Figure S5. Quantitative RT-PCR validation of expression of the top-20 tarnscriptio factors selected from the DGE analysis in *C. sieberi*. Total RNAs were extracted from yellow and white sectors of stages S6 and S8. The values are means ± SE of three independent experiments.

Table S1. Analyses of the first 100 unigenes in 8Y/8W with Log2 [fold change (FC)]> or equal to 4.1.

Name	6Y/8W	6W/8W	8Y/8W	Description
c75947_g2_i1	14,793369	1,344174	31,943395	TAIR|locus:2087418 - symbol:CCD1
c75947_g2_i2	10,433754	-1,009245	23,434988	TAIR|locus:2087418 - symbol:CCD1
c67651_g1_i1	24,778968	2,206695	22,705799	TAIR|locus:2043112 - symbol:GSTU7 "AT2G29420"
c54768_g1_i1	11,060629	1,594976	16,088108	TAIR|locus:2089055 - symbol:HS1 "heat stable protein 1"
c75947_g2_i4	7,395707	1,177903	12,69657	TAIR|locus:2087418 - symbol:CCD1
c61165_g1_i1	8,006286	1,648608	12,536966	TAIR|locus:2181793 - symbol:AT5G02550 "AT5G02550"
c71813_g3_i1	4,47089	1,521173	10,09658	TAIR|locus:2061554 - symbol:AT2G34700
c75020_g1_i1	9,265837	1,163854	9,443675	UNIPROTKB|Q9ZS29 - symbol:GAGA2
c53647_g1_i1	4,6293	4,822009	9,359946	TAIR|locus:2076969 - symbol:AT3G02630
c72390_g1_i1	4,77264	1,486662	8,774398	TAIR|locus:2097993 - symbol:AT3G62160
c48880_g2_i1	4,445774	1,227135	8,642308	UNIPROTKB|B0EXJ8 - symbol:16OMT
c73464_g2_i1	4,453448	1,669634	8,621018	TAIR|locus:2142878 - symbol:TT7
c22017_g2_i1	21,645775	3,673647	8,219751	TAIR|locus:2050901 - symbol:AT2G45180
c6283_g1_i1	6,313681	1,607327	8,200485	UNIPROTKB|Q2U0V9 - symbol:AO090011000286
c68112_g3_i1	6,986236	5,623063	7,941297	TAIR|locus:2127831 - symbol:AT4G32460
c72390_g1_i2	3,887888	-1,05576	7,471362	TAIR|locus:2097993 - symbol:AT3G62160
c72976_g7_i1	5,55582	1,321736	7,433307	TAIR|locus:505006556 - symbol:UGT73B1
c51115_g3_i1	2,416409	1,590137	7,16215	TAIR|locus:2041036 - symbol:ARA4 "AT2G43130"
c47864_g1_i1	5,844103	1,489022	6,973803	TAIR|locus:2163766 - symbol:TRY "AT5G53200"
c71470_g2_i1	3,662757	2,052728	6,726912	TAIR|locus:2175796 - symbol:AT5G60710
c68341_g1_i2	5,858407	1,458376	6,350643	TAIR|locus:2036803 - symbol:AT1G61930
c76462_g2_i1	11,392129	2,144638	6,349441	TAIR|locus:2082702 - symbol:pPLAIIIbeta "AT3G54950"
c307_g1_i1	2,452616	1,041774	6,332279	UNIPROTKB|Q75GI1 - symbol:OSJNBa0013A09.16
c76462_g2_i2	12,102115	1,937618	6,22424	TAIR|locus:2082702 - symbol:pPLAIIIbeta "AT3G54950"
c72390_g1_i3	3,407916	1,17197	6,207	TAIR|locus:2097993 - symbol:AT3G62160
c66209_g2_i2	3,436405	2,329483	6,173253	TAIR|locus:2018442 - symbol:emb2184 "AT1G75350"
c80963_g2_i4	5,578877	1,077231	6,155582	UNIPROTKB|A8QW53 - symbol:OMT3
c61931_g1_i1	3,2107	-1,050902	6,123365	TAIR|locus:2016344 - symbol:OFP14 "AT1G79960"
c68692_g3_i1	3,509593	-1,411951	6,006196	UNIPROTKB|Q9AT54 - symbol:TOGT1
c66446_g1_i1	1,014658	1,157344	5,997404	TAIR|locus:2201180 - symbol:AT1G23150
c62546_g2_i2	1,015255	1,128803	5,951851	TAIR|locus:2137574 - symbol:AT4G37850
c76454_g1_i1	2,710207	1,057281	5,851117	TAIR|locus:2120648 - symbol:sks4 "AT4G22010"
c69439_g1_i1	5,240293	1,005649	5,763792	TAIR|locus:2120683 - symbol:SRF8 "AT4G22130"
c49850_g1_i1	8,271157	2,702941	5,723581	TAIR|locus:2024967 - symbol:RTFL3 "AT1G07490"
c70157_g1_i1	4,15562	1,717557	5,680375	TAIR|locus:2018149 - symbol:ABA2 "AT1G52340"
c82451_g1_i2	4,465527	1,073432	5,617369	UNIPROTKB|Q2UEM7 - symbol:AO090026000558
c70560_g1_i2	3,973471	-1,015764	5,608133	TAIR|locus:2019195 - symbol:GASA6 "AT1G74670"
c19477_g1_i1	5,51078	1,233083	5,602283	TAIR|locus:2156529 - symbol:LATE "LATE FLOWERING"
c72611_g1_i2	3,544558	1,289541	5,582354	TAIR|locus:2142878 - symbol:TT7 "AT5G07990"
c52163_g1_i1	1,597471	1,414323	5,57497	TAIR|locus:3437527 - symbol:SULTR3;1 "AT3G51895"
c76125_g1_i1	9,566811	1,601014	5,563285	TAIR|locus:2181427 - symbol:AT5G16230
c79207_g1_i2	2,572343	1,896804	5,492887	UNIPROTKB|Q0QJI1 - symbol:DGAT1
c77436_g2_i4	4,038008	1,079908	5,432438	TAIR|locus:2082702 - symbol:pPLAIIIbeta "AT3G54950"
c74776_g3_i1	4,076491	1,489204	5,401907	TAIR|locus:2040570 - symbol:DOGT1 "AT2G36800"
c38988_g1_i1	4,632907	1,081937	5,369928	TAIR|locus:2183379 - symbol:GDH2
c75412_g1_i7	3,805312	1,562225	5,349801	TAIR|locus:2181635 - symbol:IQD11 "AT5G13460"
c80126_g1_i7	1,335329	1,440068	5,302807	TAIR|locus:2060817 - symbol:AT2G30150
c68124_g1_i1	1,582524	1,782278	5,289801	TAIR|locus:2030367 - symbol:dNK "AT1G72040"
c77436_g2_i1	5,281316	1,184957	5,208758	TAIR|locus:2082702 - symbol:pPLAIIIbeta "AT3G54950"
c75187_g1_i1	2,743479	1,222989	5,188034	TAIR|locus:2095873 - symbol:AT3G58060
c71667_g2_i1	1,503575	-1,490663	5,181843	TAIR|locus:505006632 - symbol:TUB8 "AT5G23860"
c69592_g1_i3	5,869424	2,463933	5,136058	TAIR|locus:2123261 - symbol:STOMAGEN "AT4G12970"
c72262_g1_i4	3,009898	1,004396	5,106027	UNIPROTKB|Q7XDD0 - symbol:G1L5 "Protein G1-like5"
c63460_g2_i1	3,357617	2,241402	5,076749	TAIR|locus:2152995 - symbol:AT5G41800
c72573_g2_i1	3,49217	-1,143031	5,04567	TAIR|locus:2059934 - symbol:AT2G16760
c76125_g12_i	2,803111	-1,849796	4,959311	TAIR|locus:2181427 - symbol:AT5G16230
c58302_g1_i1	2,639455	2,761598	4,929861	TAIR|locus:2088319 - symbol:IQD26 "AT3G16490"
c69189_g1_i3	3,817681	1,852595	4,923629	TAIR|locus:2012773 - symbol:ERD9 "AT1G10370"
c22676_g1_i1	4,601802	3,936056	4,904793	TAIR|locus:2091642 - symbol:GOX1 "AT3G14420"
c67272_g1_i1	4,368512	1,695318	4,865523	TAIR|locus:2169404 - symbol:PGIP2 "AT5G06870"
c78674_g7_i1	3,001232	2,728062	4,861064	TAIR|locus:2038583 - symbol:AT2G27500
c73464_g2_i2	2,359934	1,382407	4,859974	TAIR|locus:2142878 - symbol:TT7 "AT5G07990"
c53269_g1_i1	3,385037	1,201278	4,844882	TAIR|locus:2200965 - symbol:PPa1 "AT1G01050"
c73116_g1_i1	2,953706	1,160751	4,843945	TAIR|locus:2036566 - symbol:DRP4C "AT1G60500"
c6824_g1_i2	2,802685	1,167183	4,815844	TAIR|locus:2086518 - symbol:AT3G27200
c31457_g1_i2	3,23893	3,964722	4,79024	UNIPROTKB|F1NN62 - symbol:Gga.5681
c50202_g1_i1	2,302822	1,640719	4,777601	TAIR|locus:2010587 - symbol:AT1G04880
c80712_g1_i1	3,560416	1,91008	4,75016	TAIR|locus:2201272 - symbol:TKPR2 "AT1G68540"
c119270_g1_i	2,772515	3,10515	4,75003	TAIR|locus:2182978 - symbol:CHR17 "AT5G18620"
c31475_g1_i1	3,16667	2,899429	4,729783	TAIR|locus:2007332 - symbol:AT1G07270
c79634_g4_i1	2,189493	-1,230495	4,685822	TAIR|locus:2177003 - symbol:TUB6 "AT5G12250"
c53261_g2_i1	2,829312	1,188862	4,67822	TAIR|locus:2043273 - symbol:AT2G47710
c75056_g2_i3	3,273287	1,063945	4,667002	TAIR|locus:2088339 - symbol:UGT88A1
c48051_g1_i1	4,192266	1,576431	4,652978	TAIR|locus:1009023359 - symbol:AT4G14819
c71260_g1_i1	3,132967	1,124561	4,632029	TAIR|locus:2010401 - symbol:RSH2
c74094_g1_i2	2,668106	1,395764	4,618457	TAIR|locus:2032100 - symbol:GSTU19
c76335_g5_i4	2,460809	1,151138	4,604659	TAIR|locus:2165507 - symbol:TRM30
c73244_g2_i2	2,461184	1,334386	4,591772	TAIR|locus:2101948 - symbol:UGT73C7
c71488_g1_i1	4,271296	1,627268	4,583358	UNIPROTKB|Q32SG3 - symbol:LBD6
c70036_g1_i1	3,595363	1,03479	4,563596	TAIR|locus:2184133 - symbol:AT5G10150
c77698_g2_i2	1,437676	1,069921	4,562449	TAIR|locus:2150089 - symbol:LAX1 "AT5G01240"
c74936_g1_i1	2,097925	1,052181	4,547428	UNIPROTKB|P37272 - symbol:PSY1
c83544_g1_i1	3,173774	-1,255239	4,545697	TAIR|locus:2046643 - symbol:AT2G24580
c80297_g1_i1	2,286316	-1,347912	4,479776	TAIR|locus:2163951 - symbol:BGAL10 "AT5G63810"
c43677_g1_i1	3,736121	1,761553	4,469306	TAIR|locus:2007705 - symbol:AT1G48330
c61745_g1_i1	1,304996	1,360498	4,46918	TAIR|locus:2025297 - symbol:AT1G76970
c73943_g1_i1	2,529415	-1,049619	4,468196	TAIR|locus:2130359 - symbol:IAGLU
c57909_g1_i1	2,406303	-1,056183	4,452133	TAIR|locus:2194554 - symbol:XTH33 "AT1G10550"
c77439_g3_i1	2,365738	-1,257109	4,428058	UNIPROTKB|P27490 - symbol:CAB8
c68083_g1_i1	2,80466	1,396141	4,381166	TAIR|locus:2040600 - symbol:UGT73C2 "AT2G36760"
c76152_g1_i6	3,889788	1,252883	4,376977	TAIR|locus:2055007 - symbol:AT2G44940
c60353_g1_i2	4,227619	-1,06069	4,370746	TAIR|locus:2196060 - symbol:BXL2 "AT1G02640"
c79075_g2_i7	1,58282	1,055682	4,363029	UNIPROTKB|D2H6P5 - symbol:KDELC2
c73244_g1_i2	4,059137	1,89096	4,35557	UNIPROTKB|Q9AT54 - symbol:TOGT1
c80005_g2_i1	1,954203	1,00903	4,339136	TAIR|locus:2012250 - symbol:AT1G09480
c78083_g2_i1	2,109649	1,081105	4,269744	TAIR|locus:2137390 - symbol:AT4G27870
c63348_g1_i1	1,603673	1,570326	4,262653	TAIR|locus:2020153 - symbol:AT1G54200
c156940_g1_i	3,539142	1,126868	4,256812	TAIR|locus:2134796 - symbol:NCED4
c70036_g1_i4	2,5849	1,094254	4,249303	TAIR|locus:2184133 - symbol:AT5G10150
c75412_g1_i9	3,113099	1,183908	4,209477	TAIR|locus:2181635 - symbol:IQD11 "AT5G13460"

Table S2. Unigenes encoding for proteins involved in photosystems PSI and PSII, in light reactions and in photorespiration and their differential expression in the corresponding samples.

	6Y/8W	6W/8W	8Y/8W	Identity
**PSI and PSII**				
c159403_g1_i1	-1,561245	-1,400851	-2,003563	UNIPROTKB|Q6ENJ7 - symbol:psbA "Photosystem II protein D1"
c72937_g1_i1	4,842941	1,432153	2,904372	TAIR|locus:2823639 - symbol:NPQ4 "NonPhotochemical Quenching 4"
c41584_g1_i2	2,011251	1,66043	1,513342	TAIR|locus:2079117 - symbol:LHCA4 "light-harvesting chlorophyll-protein complex I subunit A4"
c81448_g8_i1	1,158003	2,076362	1,441301	UNIPROTKB|Q6ENF3 - symbol:psaJ "Photosystem I reaction center subunit IX"
c81279_g2_i4	1,252347	2,502373	1,355457	UNIPROTKB|Q1G1M8 - symbol:psaB "Photosystem I P700 chlorophyll a apoprotein A2"
c74438_g6_i1	1,403523	2,498305	1,081555	UNIPROTKB|Q6ENH5 - symbol:psaB "Photosystem I P700 chlorophyll a apoprotein A2"
c72944_g1_i3	1,088592	2,16845	-1,07357	UNIPROTKB|Q6ENG3 - symbol:ycf4 "Photosystem I assembly protein Ycf4"
c72630_g1_i1	-1,131485	-1,305262	-3,441071	TAIR|locus:2016605 - symbol:HEMA1 "AT1G58290" "protoporphyrinogen IX biosynthetic process"
**PS. Light reaction ATP synthase**				
c77290_g2_i2	1,073791	2,792732	1,355824	UNIPROTKB|Q6ENG7 - symbol:atpB "ATP synthase subunit beta, chloroplastic"
c79352_g3_i4	1,180657	2,204359	1,141968	UNIPROTKB|Q6ENH8 - symbol:atpF "ATP synthase subunit b, chloroplastic"
c79352_g3_i6	1,139859	2,193111	-1,147849	UNIPROTKB|Q6ENH9 - symbol:atpH "ATP synthase subunit c, chloroplastic"
**PS. Light reaction cytochrome b6/f**				
c76717_g1_i7	-2,158542	-2,147535	-2,115179	UNIPROTKB|Q6ENE3 - symbol:petD "Cytochrome b6-f complex subunit 4"
c72183_g6_i1	1,20912	6,073829	1,853193	UNIPROTKB|P0C319 - symbol:petD "Cytochrome b6-f complex subunit 4"
c62795_g1_i2	-1,115344	2,057191	1,243607	UNIPROTKB|Q6ENF4 - symbol:petG "Cytochrome b6-f complex subunit 5"
c65640_g2_i1	1,298823	2,024912	1,237431	UNIPROTKB|Q6ENA4 - symbol:ndhG "NAD(P)H-quinone oxidoreductase subunit 6, chloroplastic"
**PS. Calvin cycle**				
c64389_g2_i2	1,512106	1,830884	2,419792	TAIR|locus:2033686 - symbol:RBCS1A "AT1G67090" chloroplast ribulose bisphosphate carboxylase complex"
c69312_g1_i1	-3,96759	-2,043426	-1,368608	TAIR|locus:2033686 - symbol:RBCS1A "AT1G67090"chloroplast ribulose bisphosphate carboxylase complex"
c57546_g1_i1	2,265191	2,346036	2,400643	UNIPROTKB|Q43157 - symbol:RPE "Ribulose-phosphate 3-epimerase, chloroplastic"
c78320_g4_i1	1,428634	3,220507	1,898354	UNIPROTKB|Q5K3B1 - symbol:rbcL "Ribulose bisphosphate carboxylase large chain"

Table S3. Unigenes encoding for proteins involved in the starch biosynthesis pathway.

Contig	6Y/8W	6W/8W	8Y/8W	Identity
c80234_g1_i4	1,657261	-1,171096	2,671457	TAIR|locus:2036778 - symbol:GPT2 "glucose-6-phosphate/phosphate translocator 2"
c80234_g1_i5	1,412533	-1,177732	2,427693	TAIR|locus:2036778 - symbol:GPT2 "glucose-6-phosphate/phosphate translocator 2"
c74642_g1_i4	1,355248	-1,097441	2,011639	TAIR|locus:2017899 - symbol:SGB1 "AT1G79820" sugar:proton symporter activity"
c57309_g1_i1	2,474985	2,645385	1,538257	TAIR|locus:2151089 - symbol:PWD "AT5G26570" Starch-related alpha-glucan/water dikinase"
c57309_g3_i1	2,235888	1,900089	1,486078	TAIR|locus:2151089 - symbol:PWD "AT5G26570" Starch-related alpha-glucan/water dikinase"
c57309_g2_i1	1,361536	2,156934	1,170903	TAIR|locus:2151089 - symbol:PWD "AT5G26570" Starch-related alpha-glucan/water dikinase"
c82025_g1_i5	-2,019316	-1,246399	-1,699276	TAIR|locus:2151089 - symbol:PWD "AT5G26570" Starch-related alpha-glucan/water dikinase"
c79424_g5_i1	-2,433148	-1,612881	-2,260435	TAIR|locus:2148201 - symbol:AT5G17010 "sugar:proton symporter activity"
c54518_g1_i2	-2,001448	-1,635318	-1,625926	UNIPROTKB|Q01401 - symbol:SBE1 "1,4-alpha-glucan-branching enzyme, chloroplastic/amyloplastic"
c78677_g6_i2	-1,673878	-1,399946	-2,0247	TAIR|locus:2076086 - symbol:BAM1 "AT3G23920 "beta-amylase activity"
c78677_g6_i1	-1,534169	-1,440748	-2,175859	TAIR|locus:2076086 - symbol:BAM1 "AT3G23920" beta-amylase activity"
c78677_g7_i1	-1,106484	1,066509	-2,305276	TAIR|locus:2076086 - symbol:BAM1 "AT3G23920"beta-amylase activity"
c78677_g3_i1	-1,110095	-1,02612	-2,799522	TAIR|locus:2076086 - symbol:BAM1 "AT3G23920" beta-amylase activity"
c78677_g7_i5	-1,059038	1,061746	-2,907649	TAIR|locus:2076086 - symbol:BAM1 "AT3G23920" beta-amylase activity"
c78677_g7_i4	-1,107098	-1,065041	-2,921162	TAIR|locus:2076086 - symbol:BAM1 "AT3G23920" beta-amylase activity"
c78677_g7_i3	-1,110748	-1,000653	-3,233774	TAIR|locus:2076086 - symbol:BAM1 "AT3G23920"beta-amylase activity"
c81455_g2_i3	1,237456	1,375149	-4,157993	TAIR|locus:2130504 - symbol:CT-BMY "chloroplast beta-amylase"
c70427_g1_i1	-1,835802	-2,802636	-1,245441	UNIPROTKB|P53535 - symbol:STP-1 "Alpha-1,4 glucan phosphorylase L-2 isozyme, chloroplastic/amyloplastic"
c82280_g1_i2	-1,453491	-2,13847	-1,25517	TAIR|locus:2019953 - symbol:SEX1 "AT1G10760 "alpha-glucan, water dikinase activity"
c82280_g1_i1	-1,539026	-2,064989	-1,276184	TAIR|locus:2019953 - symbol:SEX1 "AT1G10760" alpha-glucan, water dikinase activity"
c54518_g1_i2	-2,001448	-1,635318	-1,625926	UNIPROTKB|Q01401 - symbol:SBE1 "1,4-alpha-glucan-branching enzyme, chloroplastic/amyloplastic"
c55442_g1_i2	-2,102263	-2,367655	1,002206	TAIR|locus:2156263 - symbol:ADG1 "AT5G48300" glucose-1-phosphate adenylyltransferase activity"
c157521_g1_i1	-1,914762	1,174115	-2,972858	TAIR|locus:2182132 - symbol:APL1 "AT5G19220 "glucose-1-phosphate adenylyltransferase activity"
c67052_g1_i1	2,4749	1,643781	1,392208	TAIR|locus:2199241 - symbol:APL2 "AT1G27680" glucose-1-phosphate adenylyltransferase activity"
c62768_g1_i1	1,639232	2,251819	1,362133	TAIR|locus:2179887 - symbol:G6PD2 "AT5G13110" glucose-6-phosphate dehydrogenase activity"
c63440_g1_i1	-2,136076	-1,328274	1,084747	TAIR|locus:2037950 - symbol:GBSS1 "AT1G32900" starch synthase activity"

Table S4. List of *C. sieberi* contigs and primer pairs used for qRT-PCR.

Contig	5′-3′ Forward primer	5′-3′ Reverse primer
c61165_g1_i1	CCAAATCGGCATCAGAGAAG	CAAAGGAACACGAACAGCAG
c47864_g1_i1	TTCACTCGTTGGTTCGCCAT	GTCGATGAACTCCCACTCGG
c75020_g1_i1	GCCTATGAATTGTCGGTGCT	TTTGGAAGCTTCTTGCTGGT
c19477_g1_i1	CCAGTCTCGTCCTCAGAAGG	CGCATCCCATTCTCTCAAAT
c76152_g1_i6	ATGCGTAACTGGGGGAAGTG	ATTAAGGTACGCCGACTGGC
c70036_g1_i1	GTTCGGCCTAACTTGCTGGA	CGAGCTCAAACAAGATCGCA
c81876_g1_i1	GTCGAGTGTTCGCAGGTACA	CATGAAGAAAGGGGATTCCA
c61931_g1_i1	CATCGCACTGTACGGTCTGT	GGAGGGGAGGGAGACATTAG
c78185_g1_i2	TTACATCATGCGGTTGGAGA	ACGCCGTCAAGAAGATCAAC
c77057_g3_i1	GTATAGAGTCCGGCGAGCTG	CCCAAGAAGACACCCAAGAA
c72262_g1_i4	GTCAGGCTCTACCTCCGTGA	ATGCGTCAAGACCAACTCCT
c79916_g2_i6	CCACGAATCCCTTGTTGTCT	GCAGAGCAATTACCCTCAGC
c67557_g1_i2	CGTCGTCCTCGGTAGAGAAG	GAGGAGATTCCACTCCCACA
c307_g1_i1	CGGACAGAATCCTCACCAGT	CCTGAAGATGACGGAAGCTC
c66216_g1_i1	ACTCGAACGAGTCGAACCAT	TCTCCTTCCACCTCCACATC
c50202_g1_i1	GAAACCACTTCACCCAGGAA	GACGTTCTTGAATCGGCACT
c82083_g1_i2	CTCCTCCGCCGTTAACTATG	ACGGGGAACAAATCAGAACA
c45682_g1_i1	GAAGGTGGAGGAGGAGGAAC	GGCTCGGAACCTTCGATACT
c79700_g2_i5	GATCATCAAAGGCAGCACCT	CTTCGGGACTAGCAGTTTGG
c67821_g2_i3	CAATCTGCATTTTGGTCGTG	GAAGCAGGTAGCATGCATCA
